# Intra- vs Intermolecular Aurophilic Contacts in Dinuclear Gold(I) Compounds: Impact on the Population of the Triplet Excited State

**DOI:** 10.1021/acs.inorgchem.2c03351

**Published:** 2022-12-13

**Authors:** Araceli de Aquino, Jas S. Ward, Kari Rissanen, Gabriel Aullón, João Carlos Lima, Laura Rodríguez

**Affiliations:** † Departament de Química Inorgànica i Orgànica, Secció de Química Inorgànica, Universitat de Barcelona, Martí i Franquès 1-11, 08028 Barcelona, Spain; ‡ Institut de Nanociència i Nanotecnologia (IN2UB), Universitat de Barcelona, 08028 Barcelona, Spain; § Department of Chemistry, Nanoscience Center, University of Jyvaskyla, 40014 Jyvaskylä, Finland; ∥ Institut de Química Teòrica i Computacional (IQTCUB), Universitat de Barcelona, 08028 Barcelona, Spain; ⊥ LAQV-REQUIMTE, Departamento de Química, Faculdade de Ciências e Tecnologia, Universidade Nova de Lisboa, 2829-516 Caparica, Portugal

## Abstract

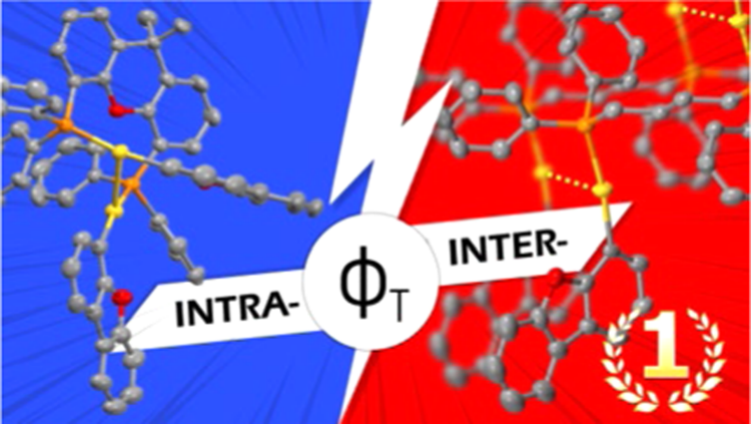

Two series of dinuclear gold(I) complexes that contain two Au–chromophore units (chromophore = dibenzofurane or dimethylfluorene) connected through a diphosphane bridge that differs in the flexibility and length (diphosphane = dppb for 1,4-bis(diphenylphosphino)butane, DPEphos for bis[(2-diphenylphosphino)phenyl]ether, xanthphos for 4,5-bis(diphenylphosphino)-9,9-dimethylxanthene, and BiPheP for 2,2′-bis(diphenylphosphino)-1,1′-biphenyl) have been synthesized and structurally characterized. Their photophysical properties have been carefully investigated, paying attention to the role of the presence, or absence, of aurophilic contacts and their nature (intra- or intermolecular character). This analysis was permitted due to the X-ray crystallographic determination of all of the structures of the compounds discussed herein. The quantum yields of the triplet population, ϕ_T_, have been calculated by nanosecond-laser flash photolysis measurements, and we could determine the main role of the character of the aurophilic contacts in the resulting ϕ_T_, being especially favored in the presence of intermolecular contacts. Time-dependent density functional theory (TD-DFT) calculations support the absorption and emission assignments and the shorter distance between S_1_ and the closest triplet excited state energy in the case of the compounds with a higher triplet-state population.

## Abstract 2

The nature of the aurophilic contacts has been determined to be a key parameter for the efficient population of the triplet excited state through intersystem crossing. This has been carefully analyzed for two series of dinuclear gold(I) compounds with two different chromophores, and we have observed that, in these cases, aurophilic intermolecular contacts favor the triplet-state population.

## Introduction

Gold(I) is known to display the highest spin–orbit coupling of the d-block metals,^1,2^ and as a consequence, it can be used as a promotor of phosphorescent emission^3−5^ due to the enhancement of the intersystem crossing transitions (S_1_ → T_1_ and T_1_ → S_0_). Once the triplet state is populated, phosphorescence emission can be observed when the excitons decay through efficient radiative T_1_ → S_0_ pathways. This phenomenon is of great relevance for the preparation of novel devices, such as PhOLEDs,^6^ which are classified into the third generation of photodevices. They are able to harvest excitons from either the singlet or triplet energy states, which makes them more efficient than previous generations.^7−9^

There are several factors that have been observed to affect the resulting luminescence of gold(I) complexes, such as the type of ligand coordinated to the metal atom through the establishment of a Au–C σ-bond.^10−13^ However, another clear factor that has been observed to affect these properties is the presence of aurophilic interactions, which can be defined as the interaction between two gold centers by intra- or intermolecular contacts^14^ with distances below the sum of their van der Waals radii (<ca. 3.5 Å).^15^ These interactions can, in some cases, favor even more the population of the T_1_ state.^16−19^

Significant effort has been made in recent years to analyze in detail the presence of these types of weak interactions,^20−23^ where there is a great necessity to investigate, predict, and/or understand their behavior and identify the structural parameters that will affect the formation of Au···Au bonds.^24,25^

To try to go one step further on the analysis of the photophysics of gold(I) compounds, in this work, a systematic analysis of the luminescent properties of two series of dinuclear gold(I) compounds has been performed. The compounds present two Au–chromophore units connected through a diphosphane bridge that differs in flexibility and length. Hence, 1,4-bis(diphenylphosphino)butane (dppb) was used to obtain flexible systems. On the other hand, the flexibility has been limited in various ways using either bis[(2-diphenylphosphino)phenyl] ether (DPEphos), 4,5-bis(diphenylphosphino)-9,9-dimethylxanthene (xanthphos) or 2,2′-bis(diphenylphosphino)-1,1′-biphenyl (BiPheP). The different structural characteristics of the diphosphanes used are expected to affect the ratio of intra- vs intermolecular gold(I) bonds.

The effect of the chromophore has also been considered, incorporating rigid molecules with planar geometry that could induce inter- or intramolecular interactions such as π···π or C–H···π.^26^ The two different chromophores chosen were dibenzofuran, well known to present thermal stability,^6,27,28^ and 9,9′-dimethylfluorene, well known to have promising potential for the preparation of deep-blue organic light-emitting diodes (OLEDs).^29−31^ They have been carefully chosen since they present similar expected quantum yield triplet formation, ϕ_T_ (0.39 for dibenzofuran and 0.22–32 for fluorene).^32^ In this way, the effect of the coordination to the gold(I) atom, the proximity of the two gold(I) units in the molecule, and the possible establishment of intra- or intermolecular aurophilic contacts can be determined. Fortunately, the X-ray crystal data for all compounds were obtained, providing relevant information about the structure (distances and angles) of the molecules and their packing in their crystalline forms. Density functional theory (DFT) calculations have also been performed to rationalize in more detail the observed transitions both in the ground and excited states and understand the differences between the calculated ϕ_T_ values.

## Results and Discussion

### Synthesis and Characterization

**Scheme 1:**
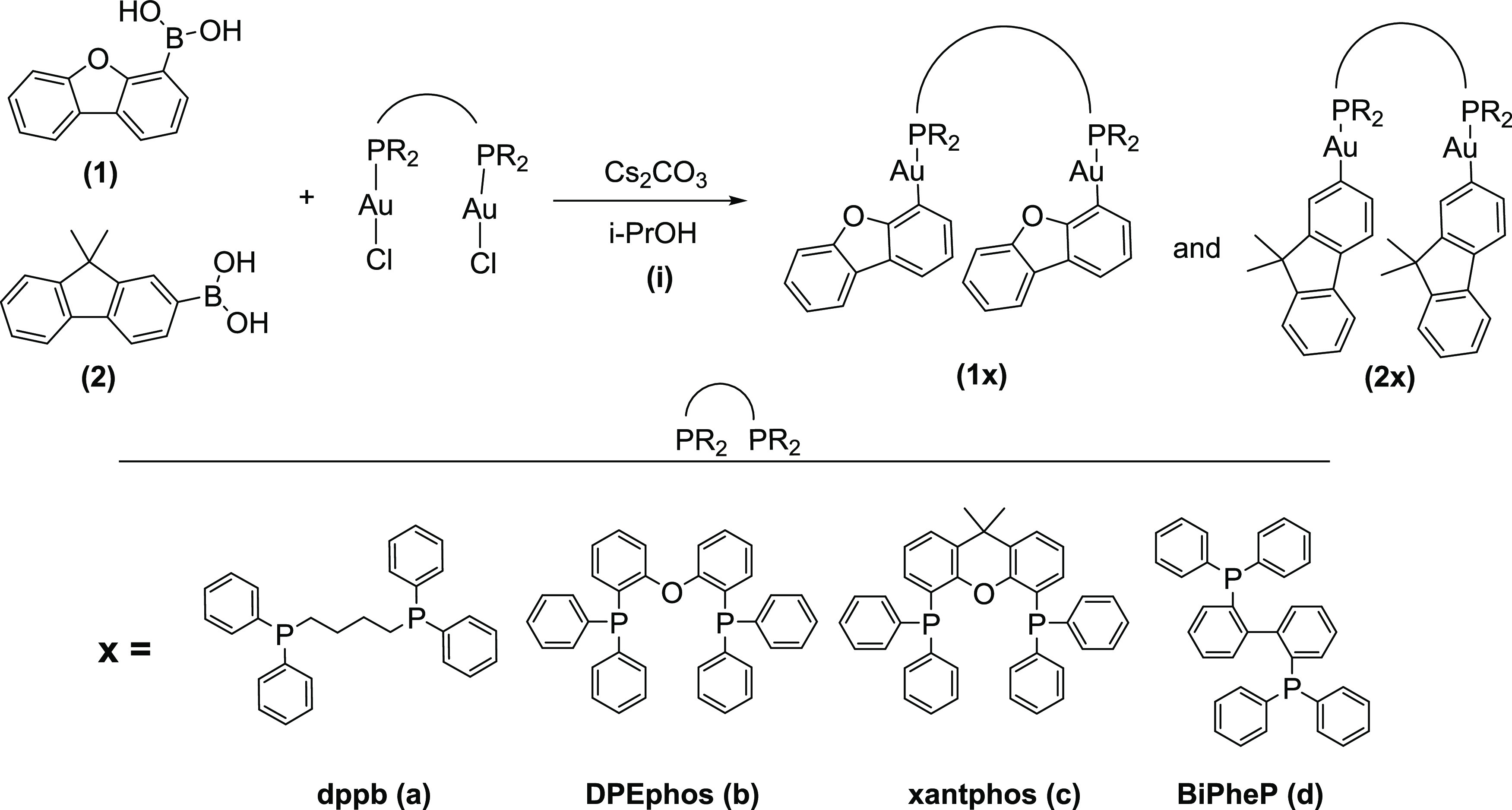
Synthesis of the Dinuclear Complexes **1a**–**d** and **2a**–**d**

Two series of dinuclear gold compounds containing a diphosphane (dppb for **1a**, **2a**, DPEphos for **1b**, **2b**, xantphos for **1c**, **2c**, and BiPheP for **1d**, **2d**) as the bridging unit and a chromophore (**1** or **2**) directly coordinated to the metal atom have been synthesized following the reaction displayed in Scheme 1. The experimental procedure was performed as previously reported by our group.^10^ That is, a solution of the boronic acid derivative of **1** or **2** with the corresponding (AuCl)_2_(diphosphane) in the presence of Cs_2_CO_3_ was heated at 50 °C (in the case of **a** and **b** diphosphane derivatives) or stirred at room temperature (RT) (for **c** and **d**) for 2 days and the final product appears as a precipitate, which was obtained in pure form after recrystallization from CH_2_Cl_2_/hexane.

All of the compounds were characterized by ^1^H and ^31^P{^1^H} NMR spectroscopy and mass spectrometry. The ^1^H NMR spectra show the signals of both the chromophore and phosphane moieties (see the Supporting Information) with the expected integration. The ^31^P{^1^H} NMR spectra show in all cases only one signal that is 20 ppm downfield shifted with respect to the (AuCl)_2_(diphosphane) precursors as an indication of the successful formation of pure products. The matrix-assisted laser desorption ionization time-of-flight (MALDI-TOF) mass spectra show the molecular peak of the compounds and peaks that belong to some fragments such as [M-fluorene]^+^, which support the successful formation of the desired compounds (Figures S1–S24).

**Figure 1:**
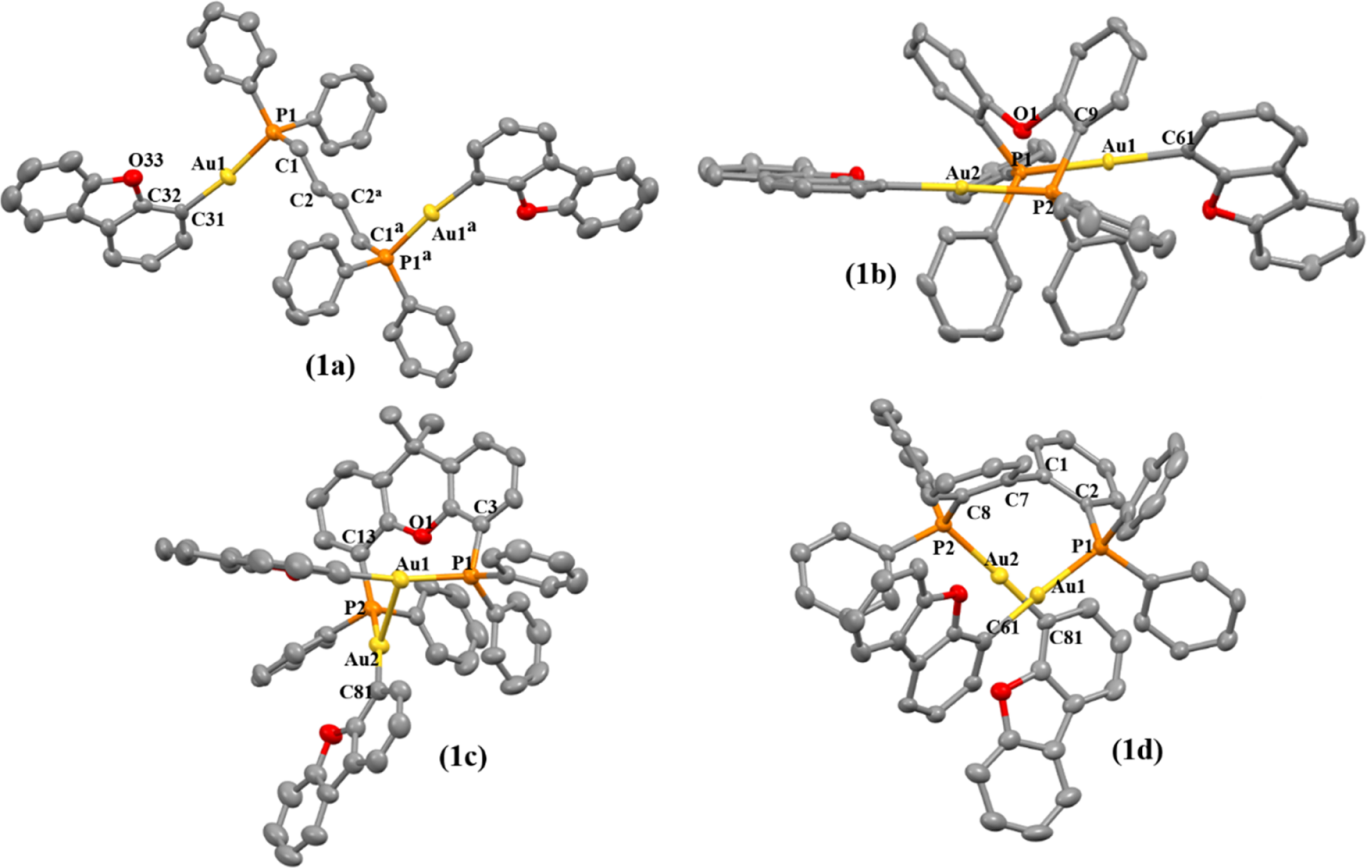
X-ray crystal structures of gold(I) compounds **1a**–**d**. Yellow, gold; orange, phosphorus; red, oxygen; gray, carbon. Thermal ellipsoids at 50% probability and hydrogen atoms were omitted for clarity.

**Figure 2:**
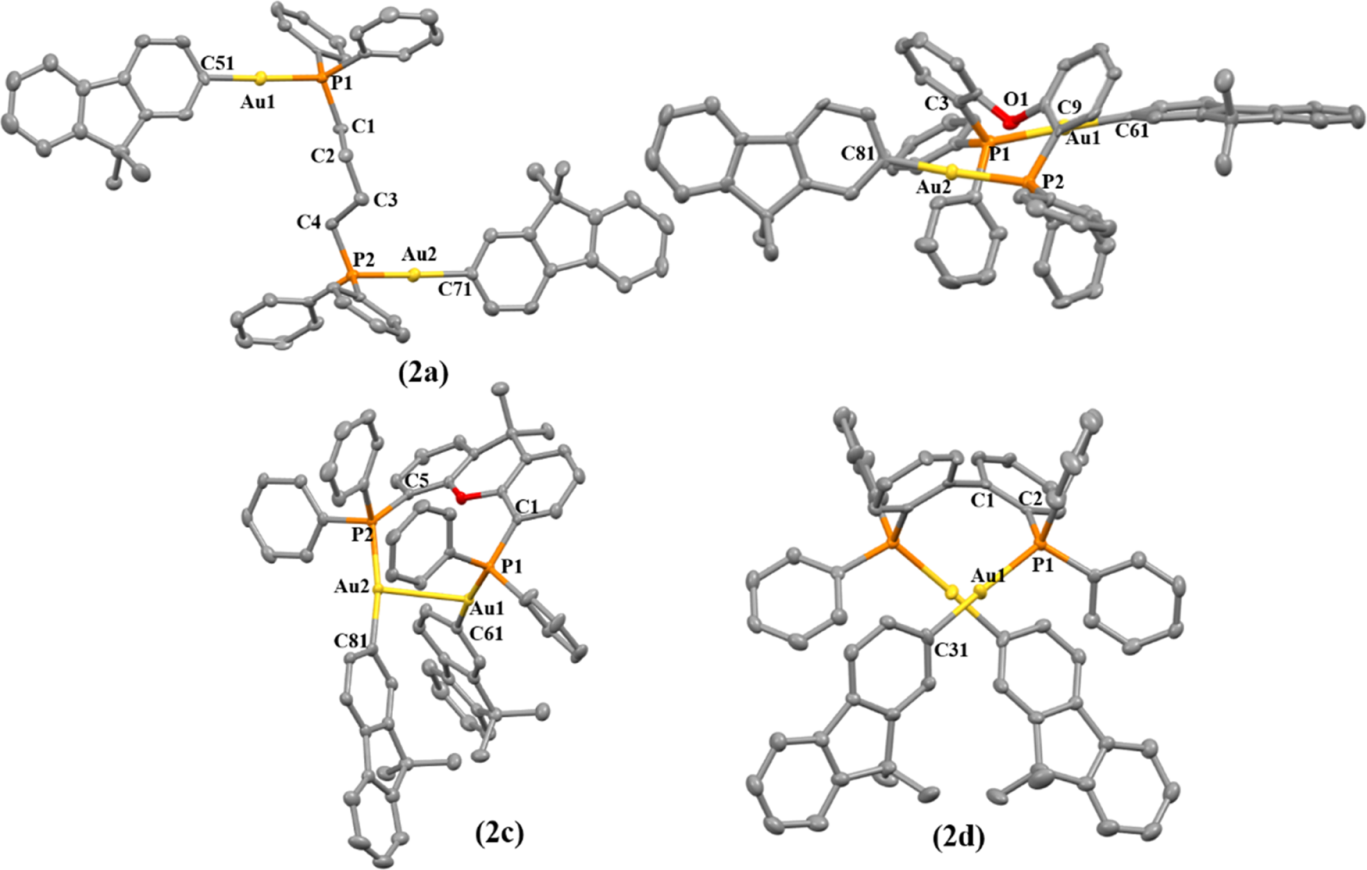
X-ray crystal structures of gold(I) compounds **2a**–**d**. Yellow, gold; orange, phosphorus; red, oxygen; gray, carbon. Thermal ellipsoids at 50% probability, and hydrogen atoms were omitted for clarity.

Single crystals suitable for X-ray diffraction analysis were successfully grown for all gold(I) compounds (Figures 1 and 2) from slow diffusion of hexane into dichloromethane solutions of the compounds at room temperature, which served as unequivocal proof of the correct formation of the desired products. The crystal data and structure refinement can be found in Tables S1 and S2, and the selected bond distances and angles are displayed in Tables 1 and 2.

**Table 1:** Selected Bond Lengths (Å) and Angles (deg) for Complexes **1a–d**

**1a**		**1b**		**1c**		**1d**	
Au1···Au2	3.4494(4)	Au1···Au2	6.182(1)	Au1···Au2	2.931(6)	Au1···Au2	3.567(1)
P1–Au1	2.287(1)	P1–Au1	2.278(9)	P1–Au1	2.301(2)	P1–Au1	2.289(1)
Au1–C31	2.084(5)	Au1–C61	2.050(3)	Au2–C81	2.048(5)	Au1–C61	2.054(7)
P1–C1	1.835(6)	P2–C9	1.821(3)	P1–C3	1.829(4)	P1–C2	1.821(6)
P1–Au1–C31	173.2(1)	P1–Au1–C61	177.22(9)	P2–Au2–C81	164.6(2)	P1–Au1–C61	176.7(2)

**Table 2:** Selected Bond Lengths (Å) and Angles (deg) for Complexes **2a**–**d**

**2a**		**2b**		**2c**		**2d**	
Au1···Au3	3.520(3)	Au1···Au2	6.205(2)	Au1···Au2	2.939(1)	Au1···Au2	3.601(8)
P1–Au1	2.300(2)	Au1–P1	2.292(2)	P1–Au1	2.301(2)	Au1–P1	2.284(2)
Au1–C51	2.072(6)	P1–C3	1.811(5)	Au2–C81	2.048(5)	Au1–C31	2.052(6)
P1–C1	1.828(8)	Au1–C61	2.042(7)	P1–C3	1.829(4)	P1–C2	1.828(5)
P1–Au1–C51	177.2(2)	P1–Au1–C61	176.3(2)	P2–Au2–C81	164.6(2)	P1–Au1–C31	177.9(2)

The coordination of the gold(I) moiety to the fluorene is linear with slightly distorted P–Au–C angles from 177 to 173° for the dppb (**1a**, **2a**), DPEphos (**1b**, **2b**), and BiPheP (**1d**, **2d**) derivatives, while the highest deviation was observed for the xantphos complexes (**1c**, **2c**) with 164.6(2)°, though with all of the values in the expected ranges for these types of compounds.^33−36^ Close intramolecular aurophilic contacts were detected for **1c**, **1d**, **2c** and **2d**, with intermolecular aurophilic interactions observed for **1a** and **2a**. That is, the more flexible dppb diphosphane favored intermolecular aurophilic interactions with respect to the more rigid diphosphane derivatives, which possessed intramolecular aurophilic bonds. These contacts have been studied further due to their relevance in their resulting photophysical properties (see below).^37,38^

#### Analysis of the Aurophilic Interactions in the X-ray Crystal Structures

The analysis of the X-ray crystal structures of the compounds provides important information regarding the establishment of weak interactions both in an intra- and intermolecular way. In general, π···π and C–*H*···π intermolecular contacts have been detected in the packing of all of the compounds. On the other hand, the different chemical structure of the diphosphanes plays a key role in the resulting intra- and intermolecular contacts due to their differing rigidities and intramolecular distances between the two P atoms. These features can induce the establishment of metallophilic interactions, with the resulting Au···Au distances, if present, being directly affected by the structure of the diphosphanes. Special attention has been paid to these types of contacts in this work.

**Figure 3:**
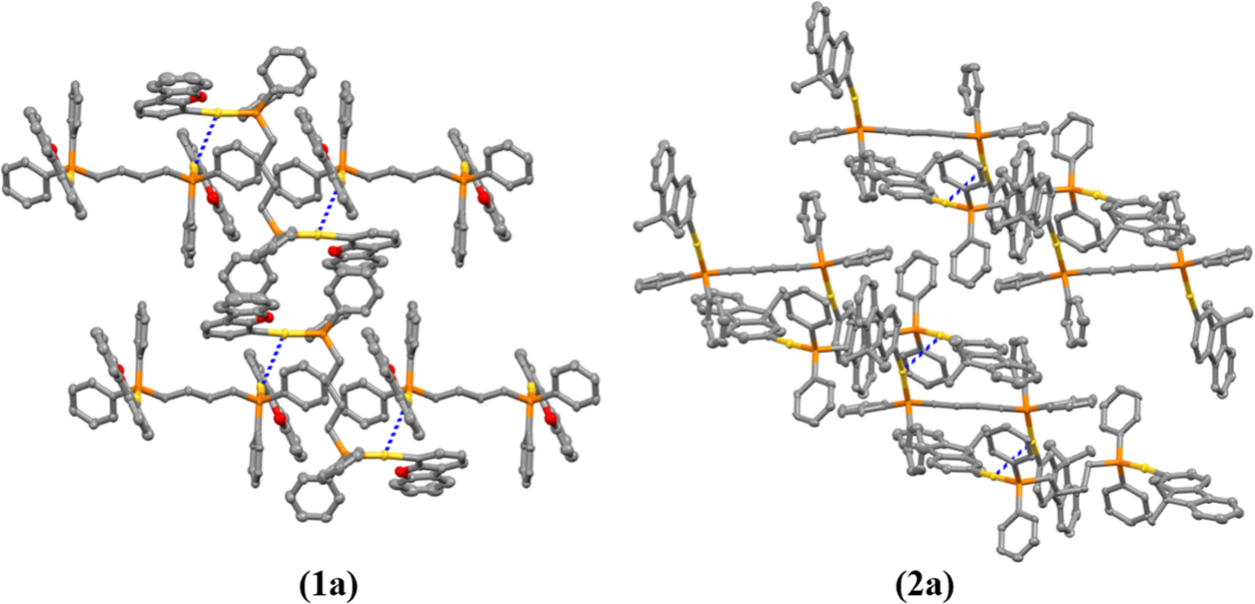
Three-dimensional (3D) crystal packing of complexes **1a** (left) and **2a** (right) viewed by the *b*-axis. Aurophilic interactions are marked with blue dotted lines.

Intermolecular aurophilic interactions have been detected for the dppb derivatives (**1a**, **2a**) with distances of 3.4494(4) and 3.5203(5) Å, respectively (Figure 3).

**Figure 4:**
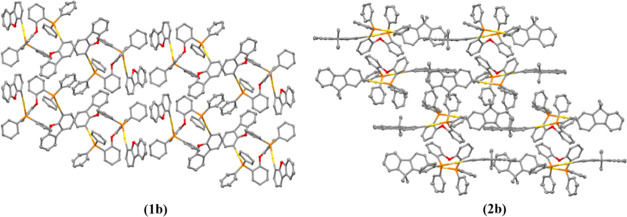
3D crystal packing of complexes **1b** and **2b** viewed down the b*-axis. Hydrogen atoms were omitted for clarity.

DPEphos derivatives **1b** and **2b** present neither intra- nor intermolecular aurophilic interactions, which can probably be ascribed to the flexibility that comes from the central oxygen atom enabling rotation of the two gold(I)–chromophore arms, which does not encourage any aurophilic contacts (Figure 4).

**Scheme 2:**
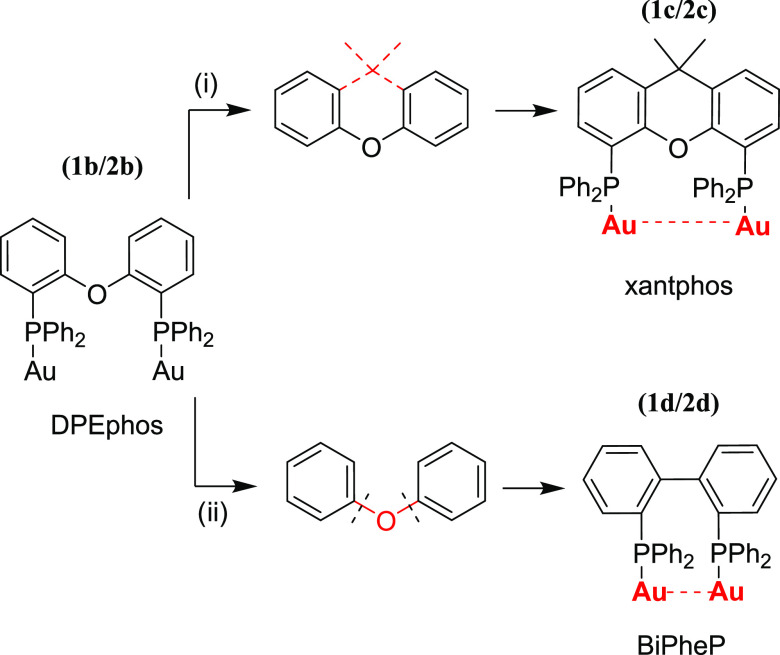
Comparison of the Rigidity of the **1b**/**2b** and **1c**/**2d** Diphosphane Units

To promote the aurophilic interactions, we tried to modulate the rigidity of this diphosphane using two different diphosphanes: (i) xanthphos (compounds **1c**, **2c**), which has a more rigid core with respect to DPEphos (Scheme 2, i), and (ii) BiPheP (compounds **1d**, **2d**), where the central oxygen atom is removed and both phenyl rings are directly connected to each other as a bridging unit between the two phosphorus atoms of diphosphane (Scheme 2, ii).

**Figure 5:**
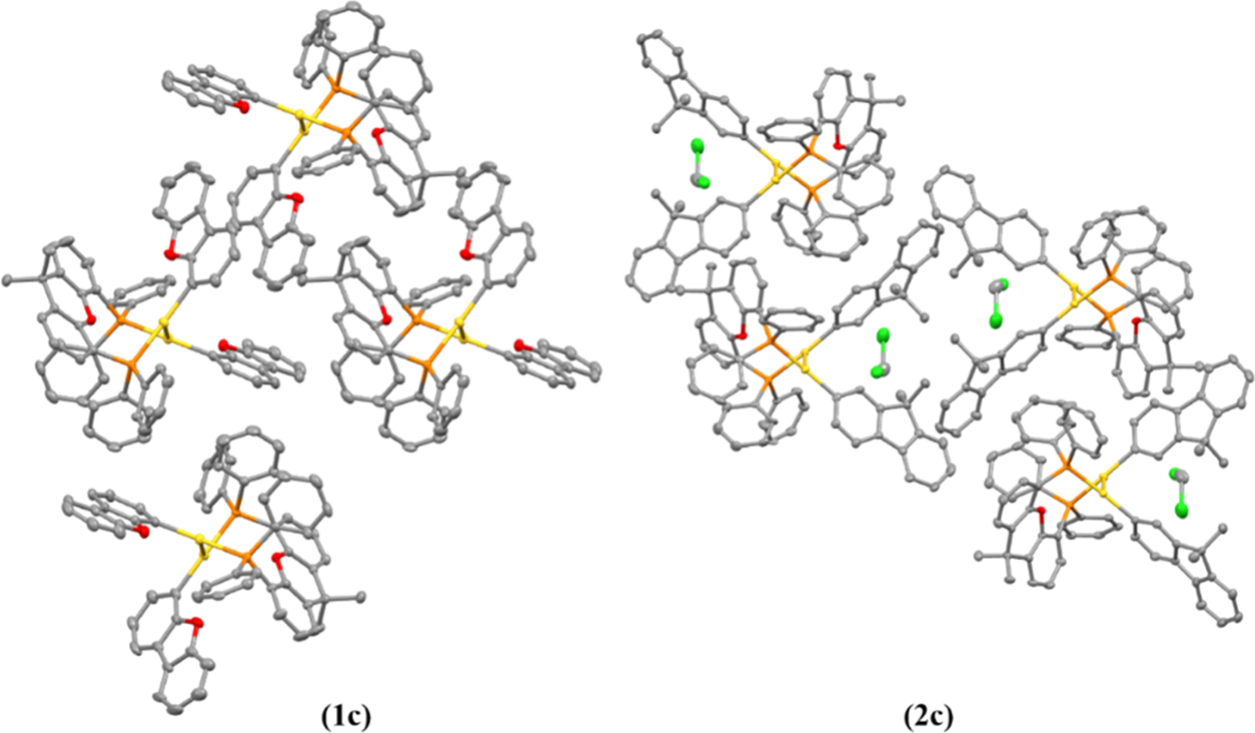
3D crystal packing of complexes **1c** and **2c** viewed down the *a*-axis. Hydrogen atoms were omitted for clarity.

Intramolecular aurophilic interactions with short distances of 2.931(6) and 2.939(1) Å (Figure 5 below) have been detected for the **1c** and **2c** derivatives, respectively, indicating the presence of very strong Au···Au contacts.

**Figure 6:**
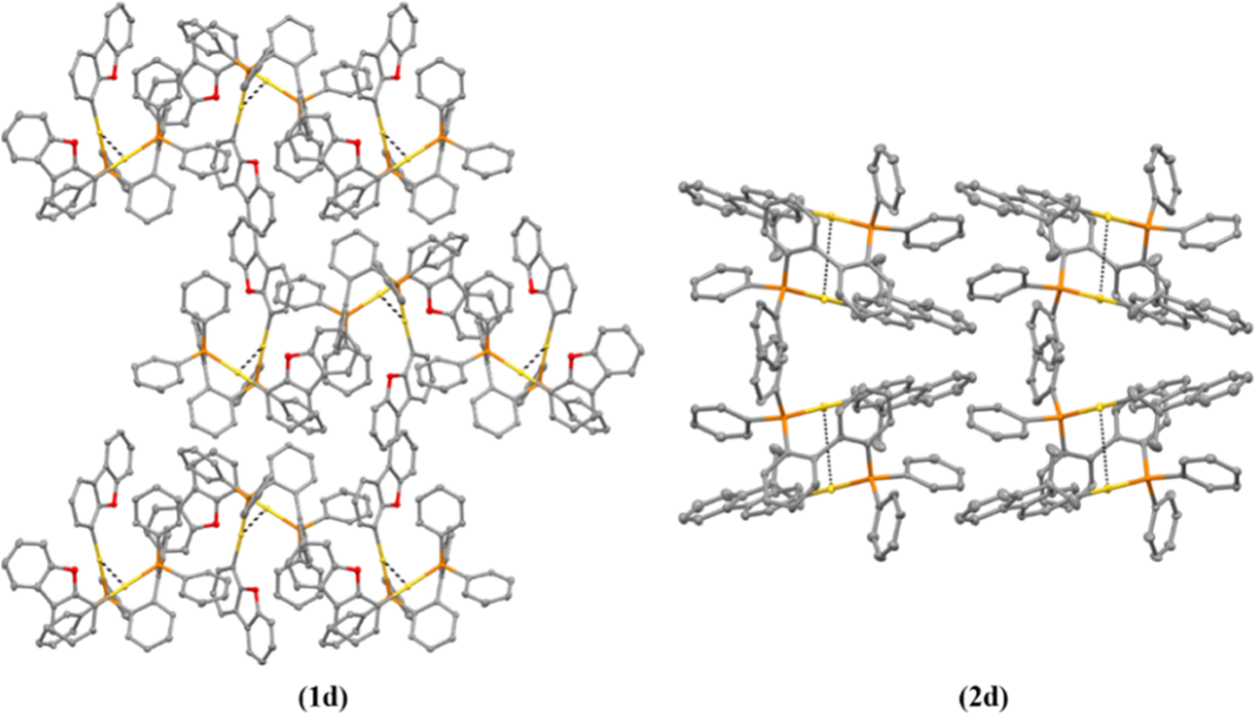
3D crystal packing of complexes **1d** and **2d** viewed down the *a*-axis (**1d**) and *b*-axis (**2d**). Aurophilic interactions are marked with black dotted lines. Hydrogen atoms were omitted for clarity.

Weak intramolecular aurophilic interactions of 3.567(1) and 3.601(8) Å were found (Figure 6) for **1d** and **2d**, respectively, probably due to the more restricted rotation in these BiPheP derivatives with respect to the DPEphos analogues.

The *d*(Au–Au) distances calculated from the X-ray crystal structure of our molecules have been used to estimate the strength of the Au···Au interaction through the formula
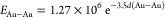
1where *E* is the energy in kJ/mol and *d*(Au–Au) is the distance between gold(I) centers in angstroms,^39^ with the results summarized in Table 3. As expected, the xantphos derivatives show the strongest aurophilic contacts with 44.4 kJ/mol (**1c**) and 43.3 J/mol (**2c**). These interactions are stronger than the more common energies found in the literature, which be present also in solutionare around 20 kJ/mol.^40−42^ Additionally, the intermolecular interactions found in the dppb derivatives **1a** and **2a** are stronger than those displayed intramolecularly by the BiPheP derivatives **1d** and **2d**. Thus, there is not a direct correlation between the intra- or intermolecular character and their strength. Finally, we can observe that the Au···Au energies calculated for series **2** compounds are slightly lower than those for series **1**, and thus, the chromophore has some small influence on the strength of these contacts.

**Table 3:** Calculated Aurophilic Energies by Equation 1

compound	*E*_Au–Au_ (kJ/mol)
**1a**	7.3
**1b**	–a
**1c**	44.4
**1d**	4.8
**2a**	5.7
**2b**	–a
**2c**	43.3
**2d**	4.3

a No aurophilic interactions were seen in the X-ray crystal structure.

### Photophysical Characterization

The absorption and emission spectra of all of the compounds **1**–**2**, **1a**–**d**, and **2a**–**d** were recorded in 1 × 10^–5^ M dichloromethane solutions at room temperature. The obtained data are summarized in Table 4.

**Table 4:** Absorption and Emission Data of **1** and **2** and Compounds **1a**–**d** and **2a**–**d** in Dichloromethane at 1 × 10^–5^ M

compound	absorption λ_max_ (nm) (10^4^ ε (M^–1^ cm^–1^))	fluorescence emission, λ_exc_ = 288 nm (solution, λ_max_ (nm)) at RT	phosphorescence emission, (λ_exc_ = 288 nm solution, λ_max_ (nm)) at 77 K
**1**	288 (1.3), 310 (0.8)	327	329
**1a**	288 (2.7), 310 (0.9)	328	414, 442
**1b**	288 (2.6), 310 (1.1)	328	413, 440
**1c**	288 (2.8), 310 (1.2)	328	414, 443
**1d**	288 (2.8), 310 (1.3)	330	417, 440
**2**	290 (1.6), 313 (1.9)	324	323
**2a**	290 (3.3), 313 (4.1)	328	440, 474
**2b**	290 (3.0), 313 (3.7)	324	441, 473
**2c**	290 (3.4), 315 (3.1)	326	445, 477
**2d**	290 (3.6), 318 (4.1)	326	450, 481

**Figure 7:**
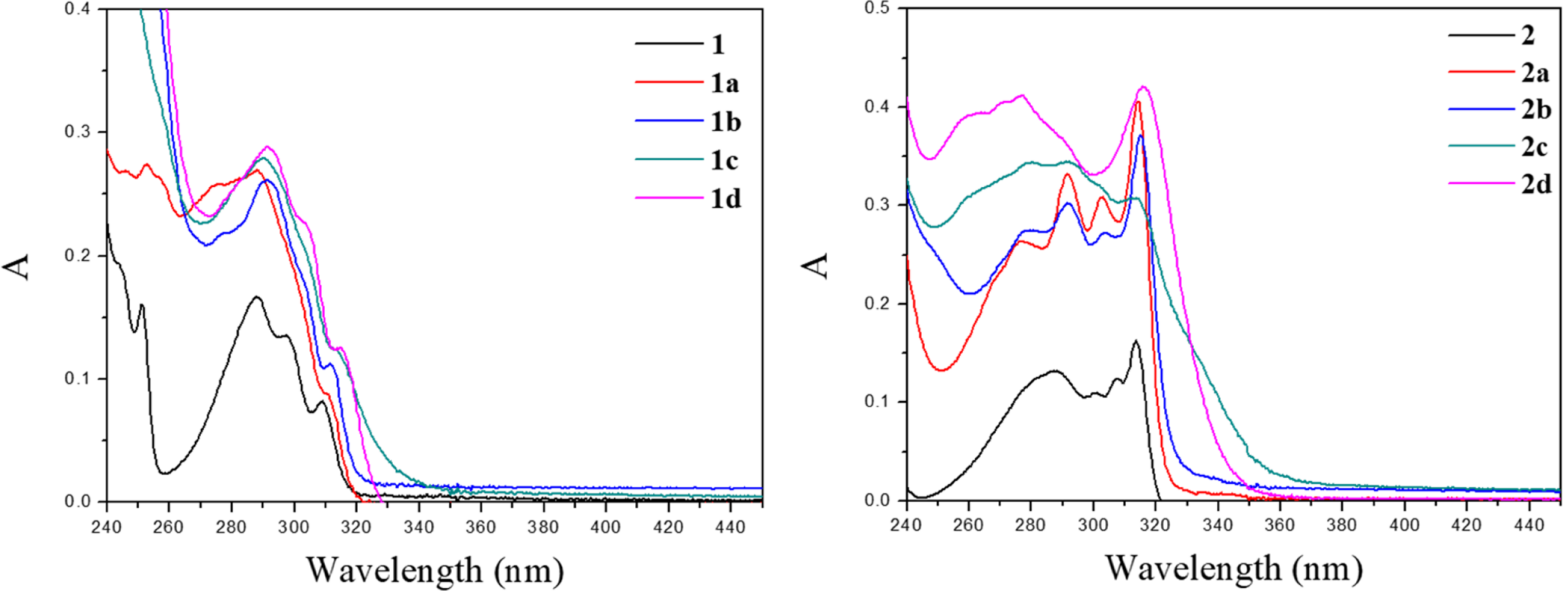
Absorption spectra of 1 × 10^–5^ M dichloromethane solutions of **1** (left) and **2** (right) derivatives.

The electronic spectra of all of the complexes (Figure 7) show two intense bands at ca. 288 and 310 nm, and the transitions can be assigned to ligand-centered π–π* transitions according to the literature.^6,28,29^ Inspection of Figure 7 shows that **1c** and **2c** compounds display an extensive broadening of the absorption bands, which is expected since the restricted geometry is kept in solution and forces the presence of Au···Au and π···π interactions (between the fluorene chromophores). Additional inspection of spectra of compounds **1d** and **2d** show that these compounds also display significant broadening, which is an indication of the proximity between the fluorene chromophores since the broadening is a consequence of the exciton splitting due to π–π stacking. Dilution of the solutions of compounds **1d** and **2d** does not eliminate the broadening, which indicates intramolecular Au···Au and π···π interactions (independent of concentration, see Figure S26).

Compounds **1a** and **2a** do not show the spectral broadening indicative of π···π exciton splitting of the chromophores, which implies the absence of Au–Au intramolecular contacts, but does not exclude Au···Au intermolecular contacts since in this case the molecules can approach in a staged conformation where the π···π interactions are absent and the Au···Au interactions are present. We have previously shown that the presence of Au···Au interactions leads to the appearance of new electronic transitions from the σ*(Au···Au) orbital to π* orbitals of the ligands.^22^ As such, we conducted experiments in compounds **1a** and **2a** by changing the concentration between 5 × 10^–6^ and 4 × 10^–5^ M and comparing the normalized spectra (Figure S25). There is clear evidence for the appearance of additional bands that affect the ratios of absorbance maxima with increasing concentration. This agrees with the appearance of new transitions due to the intermolecular contacts (aurophilic aggregates) that must be present also in the solution. Interestingly, similar studies for compounds **1b** and **2b** do not show the appearance of new transitions affecting the absorption ratios of the bands with concentration (Figure S25). The global explained variations in the absorption shape bands due to aggregation with concentration that evidences the presence of intermolecular aurophilic contacts and the maintenance of intramolecular aurophilic contacts are better represented in Figure S26.

**Figure 8:**
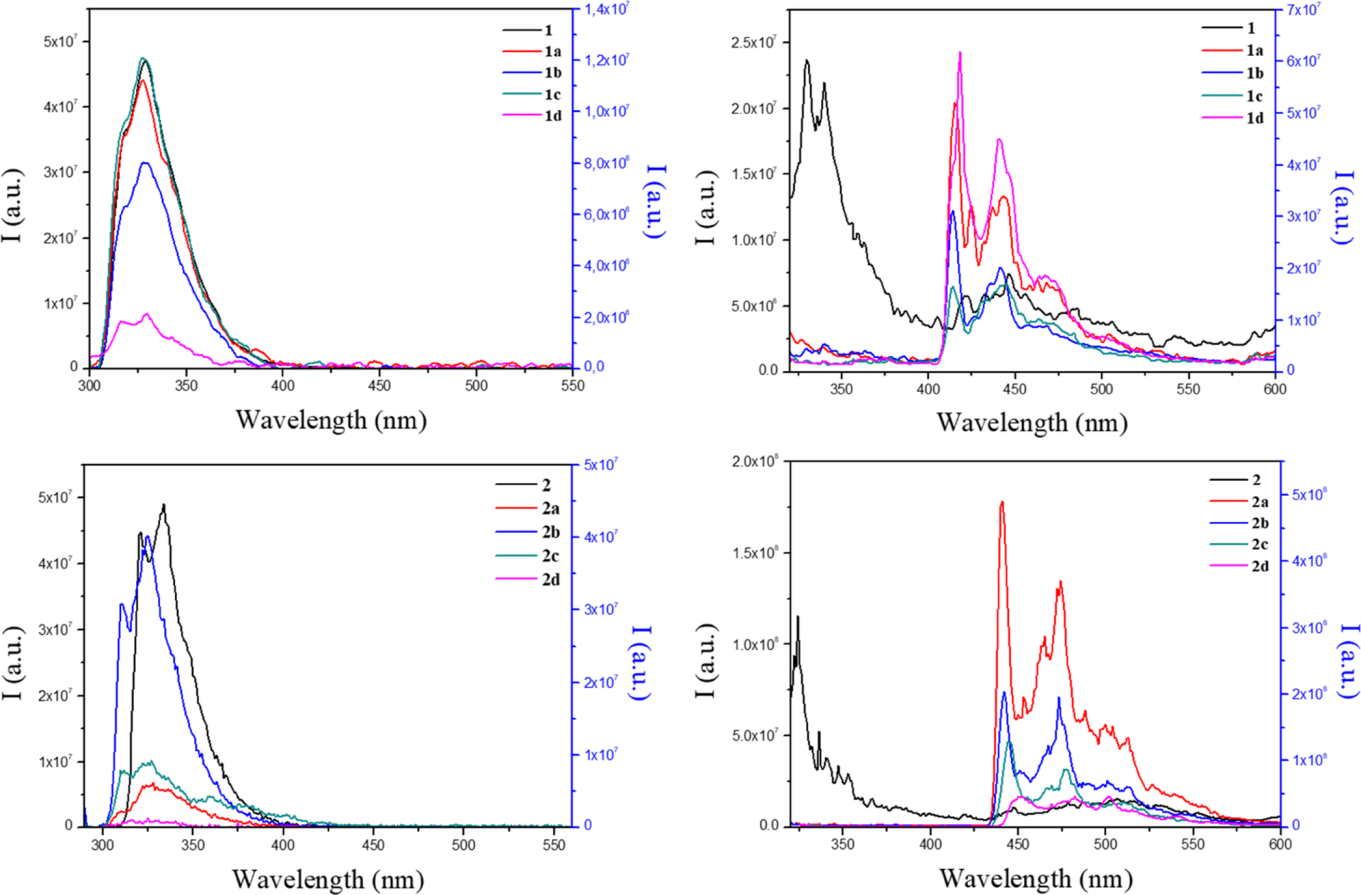
Emission spectra of 1 × 10^–5^ M solutions of all of the compounds in dichloromethane at room temperature (left) and at 77 K (right) for series **1** derivatives (above) and series **2** derivatives (below). λ_exc_ = 288 nm. Left axis, emission intensity of **1** or **2**; right axis, emission intensity of gold(I) complexes.

All compounds display a well-resolved emission band centered at ca. 330 nm when the samples were excited at λ_exc_ = 288 nm (Figure 8). The intensity of the fluorescence emission of the gold(I) compounds decreases in comparison with that of the free ligand, as expected for an efficient intersystem crossing induced by the coordination of a gold(I) atom to the organic ligand.^11^ Phosphorescence emission can be detected at ca. 450 nm for series **1** and at ca. 475 nm for series **2** when decreasing the temperature of the solution to 77 K, leading to a minimum fluorescence contribution in the emission (Figure 8).

The luminescence quantum yields in the solution have been recorded at room temperature (Table 5). The series **2** compounds display lower fluorescence quantum yields in comparison with series **1** compounds and even more considering the larger quantum yield of the organic counterpart in **2** with respect to **1**. At least in the case of compounds **1a** and **2a**, the decrease in fluorescence quantum yield is partially related to the increase in the population of the triplet, but the increase in other nonradiative channels of the singlet excited state deactivation is clearly involved here.

**Table 5:** Fluorescence (ϕ_Fl_) and Triplet (ϕ_T_) Quantum Yield Values of Compounds **1**, **1a**–**d**, **2**, and **2a**–**d** in Dichloromethane at Room Temperature

compound	ϕ_Fl_	ϕ_T_
**1**	0.30	0.17
**1a**	0.04	0.30
**1b**	0.07	0.23
**1c**	0.07	0.16
**1d**	0.02	0.22
**2**	0.83	0.27
**2a**	0.03	0.56
**2b**	0.03	0.24
**2c**	0.02	0.26
**2d**	0.02	0.13

#### Nanosecond Transient Absorption

The transient decays at 280 nm (ground-state absorption) and 450 nm (triplet-state absorption) for compounds **1**, **2**, **1a**–**d**, and **2a**–**d** in dichloromethane solutions enabled the determination of the quantum yields for triplet formation, Φ_T_ (see values in Table 5).

The Φ_T_ values of the dppb derivatives (**1a** and **2a**) are the largest among the others. The main difference resides in the fact that in the case of compounds with intramolecular aurophilic contacts the decrease in Φ_Fl_ is not reflected in an increase in Φ_T_, so the presence of aurophilic bonds adds an additional nonradiative channel to deactivation of the singlet excited state that competes with the intersystem crossing to the triplet state. This channel seems to be less effective in the case of **1a** and **2a**, where an increase in the Φ_T_ values is observed.

#### Theoretical Calculations

TD-DFT calculations were performed for all of the compounds to optimize the geometries without restraints, and the harmonic frequency calculations found the converged structures as a potential-energy minimum. Calculations were done by including continuum solvation in dichloromethane with the main goal of identifying the lower singlet and triplet states of the molecules.

All complexes present monoexcitations involving the highest occupied molecular orbital (HOMO) and the lowest unoccupied molecular orbital (LUMO). Figures S27–S34 show these molecular orbitals for the **1x** and **2x** family of compounds, and Tables 6 and S3–S5 summarize the obtained results.

**Table 6:** Calculated Lower Singlet and Triplet Energies by TD-DFT for **1a** and **2a** and Their Main Contribution in %

compound	energy (*S*_*n*_) (eV)	transitions	energy (*T*_*n*_) (eV)	transitions
**1a**	*S*_1_: 4.2878	H – 2 → L (32%)	*T*_11_: 4.2119	H – 1 → L + 14 (27%)
	*f* = 0.1598	H – 2 → L + 3 (24%)		H – 6 → L + 14 (8%)
		H → L (14%)		H – 3 → L + 14 (8%)
		H → L + 3 (11%)		
	*S*_2_: 4.2988	H – 3 → L + 2 (58%)	*T*_12_: 4.2133	H → L + 15 (27%)
	*f* = 0.1044	H – 1 → L + 2 (21%)		H – 2 → L + 16 (8%)
				H – 2 → L + 3 (8%)
			*T*_2_: 3.1895	H → L + 3 (23%)
				H → L (14%)
				H – 2 → L + 3 (9%)
			*T*_1_: 3.1891	H – 1 → L + 2 (55%)
				H – 3 → L + 2 (8%)
**2a**	*S*_1_: 4.039	H → L (43%)	*T*_10_: 3.9597	H → L + 10 (23%)
	*f* = 0.9883	H – 1 → L (31%)		H – 1 → L + 10 (18%)
		H – 1 → L + 1 (15%)		H – 5 → L + 17 (4%)
	*S*_2_: 4.0598	H → L + 1 (38%)	*T*_9_: 3.9594	H – 1 → L + 11 (26%)
	*f* = 0.4183	H – 1 → L (25%)		H → L + 11 (20%)
		H – 1 → L + 1 (19%)		H – 4 → L + 11 (8%)
		H → L (7%)		
	*S*_3_: 4.1589	H → L + 2 (43%)	*T*_2_: 3.0121	H → L + 1 (14%)
	*f* = 0.1083	H – 1 → L + 2 (34%)		H – 1 → L + 1 (11%)
		H → L (11%)		H → L (3%)
			*T*_1_: 3.0108	H – 1 → L (11%)
				H → L (9%)
				H – 1 → L + 1 (4%)

**Scheme 3:**
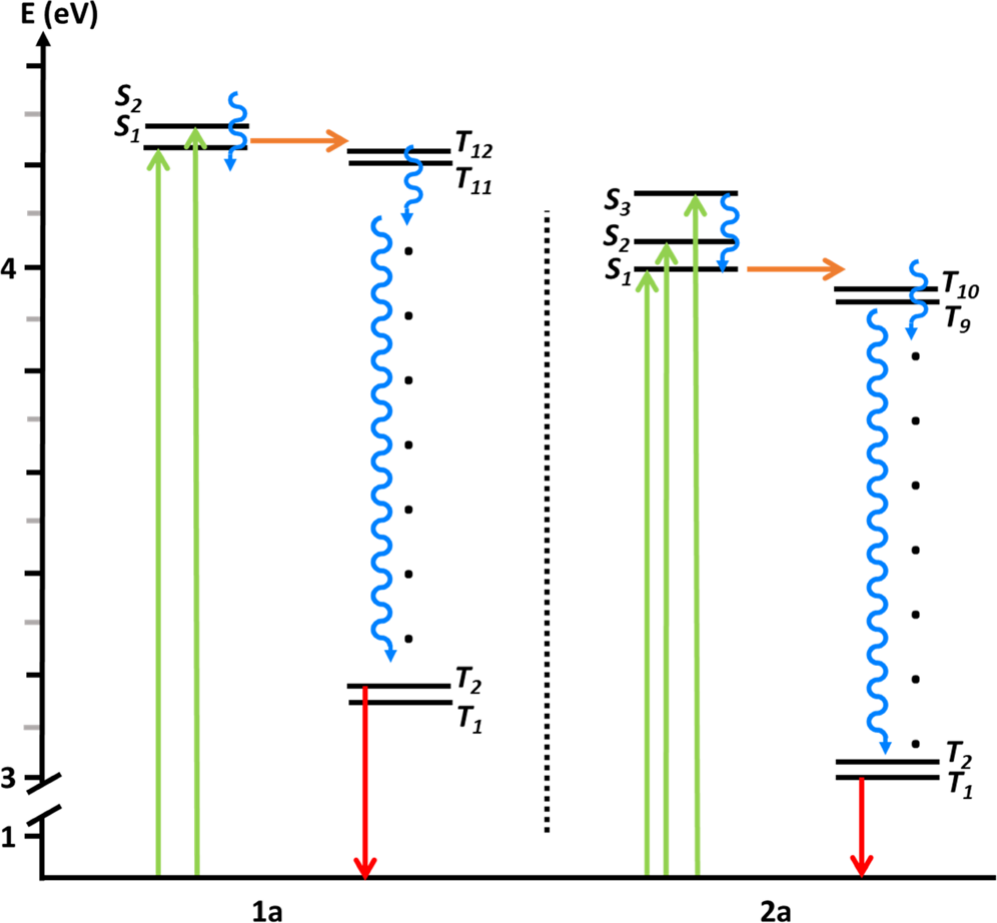
TD-DFT State Plot Showing the Energies of the Singlet and Triplet States in Electronvolts Green line, absorption; blue line, vibrational relaxation; orange line, intersystem crossing; red line, phosphorescent emission decay.

The intersystem crossing is most probably occurring between *S*_1_ and a *T*_*n*_ state with an appropriate energy and symmetry (Tables 6 and S3–S5), followed by fast relaxation to *T*_1_ within the triplet manifold (see Scheme 3).

Nevertheless, the presence of intramolecular or intermolecular Au···Au interactions can modify the intersystem crossing and also the nonradiative processes. This is evidenced by the experimental results with **1c**, **1d**, **2c**, and **2d**, where the presence of Au···Au interaction evidenced by the observed broadening in the absorption spectra does not result in the expected increase in the triplet formation quantum yield when compared with the ligand. This is probably due to the competing nonradiative process interplay that is also introduced by the excitonic interactions.

## Conclusions

The nature of the aurophilic contacts has been determined to be a key parameter for the efficient population of the triplet excited state through intersystem crossing. The studies carried out herein demonstrate that intermolecular aurophilic interactions are much more favorable toward increasing the ϕ_T_ value (efficient population of the triplet excited state), while intramolecular aurophilic contacts have a greater effect on the nonradiative deactivation channels. These results can be ascribed by the analysis of two families of dinuclear gold(I) compounds that contain two different chromophores with similar expected ϕ_T_ values (or the pure organic counterpart) but differ in the bridging ligand that connects the two gold(I) atoms. The solid-state structures were determined for all compounds by single-crystal X-ray diffraction, enabling a complete understanding of the type and proximity of all of the weak interactions involved in the packing of these compounds.

## Experimental Section

### General Procedures

All manipulations have been performed under prepurified N_2_ using standard Schlenk techniques. All solvents have been distilled from appropriate drying agents. Commercial reagents dibenzofuran-4-boronic acid, 9,9-dimethyl-9*H*-fluoren-2-ylboronic acid, bis[(2-diphenylphosphino)phenyl]ether, and 4,5-bis(diphenylphospheno)-9,9-dimethylxanthene were purchased from Fluorochem; dppb and cesium carbonate were purchased from Sigma-Aldrich; and 2,2′-bis(diphenylphosphino)-1,1′-biphenyl was purchased from Abcr.

### Physical Measurements

Infrared spectra have been recorded on a Fourier transform infrared (FT-IR) 520 Nicolet Spectrophotometer. ^1^H NMR (δ(TMS) = 0.0 ppm) and ^31^P{^1^H} NMR (δ(85% H_3_PO_4_) = 0.0 ppm) spectra have been obtained on a Varian Mercury 400 and Bruker 400, respectively. ES(+) mass spectra were recorded on a Fisons VG Quatro spectrometer. Absorption spectra have been recorded on a Varian Cary 100 Bio UV spectrophotometer, and emission spectra have been recorded on a Horiba Jobin-Yvon SPEX Nanolog spectrofluorimeter.

Transient absorption experiments were measured with a laser flash photolysis LK60 Applied Photophysics system in absorption mode after laser pulse excitation at 266 nm at the Departamento de Quimica-Universidade Nova de Lisboa. The transient decays at 280 nm (ground-state absorption) and at 450 nm (triplet-state absorption) for compounds **1**–**2**, **1a**–**d**, and **2a**–**d** in dichloromethane solutions were acquired to determine the quantum yields for triplet formation (Φ_T_).

For that, we first calculated the ε_T_ using eq 2 with the experimental ε_S_ (see Table 4) and the optical density amplitude (Δ_OD1_ belongs to the amplitude of the depletion experiment and Δ_OD2_ to the amplitude from the transient experiment), which can be calculated by the fitting using a monoexponential equation
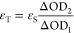
2

After that, we could know the Φ_T_ thanks to the “singlet-depletion” method (eq 3) using benzophenone as the reference actinometer
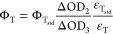
3optically matching a benzophenone solution in acetonitrile (Φ_T_ = 1; ε_T_std__ = 6500 cm^–1^ M^–1^ at 520 nm) with the absorption samples at the laser excitation wavelength (266 nm). The extinction coefficient of the triplet–triplet absorption at 450 nm was calculated from depletion at 280 nm using the extinction coefficient of the ground-state absorption at that wavelength, ε_T_, previously measured.

### Crystal Data

Single-crystal X-ray data for **1d** and **2c** were measured using a Rigaku SuperNova dual-source Oxford diffractometer equipped with an Atlas detector using mirror-monochromated Cu Kα (λ = 1.54184 Å) radiation. Single-crystal X-ray data for **1a**, **1b**, **2a**, **2b**, and **2d** were measured using a Rigaku SuperNova Oxford diffractometer equipped with an Eos detector using mirror-monochromated Mo Kα (λ = 0.71073 Å) radiation. The data collection and reduction were performed using program CrysAlisPro.^43^ Single-crystal X-ray data for **1c** was measured using a Bruker Nonius Kappa CCD diffractometer with an APEX-II detector and graphite-monochromatized Mo Kα (λ = 0.71073 Å) radiation. Data collection and reduction were performed using programs COLLECT^44^ and HKL DENZO AND SCALEPACK,^45^ respectively, and the intensities were corrected for absorption using SADABS.^46^ All structures were solved by intrinsic phasing (SHELXT)^47^ and refined by full-matrix least squares on *F*^2^ using OLEX2 software^48^ utilizing the SHELXL module.^49^ CCDC 2193612–2193619 contain the supporting crystallographic data for these structures. These data can be obtained free of charge from The Cambridge Crystallographic Data Centre via www.ccdc.cam.ac.uk/data_request/cif.

### Synthesis and Characterization

#### Synthesis of **1a**

Dibenzofuran-4-boronic acid (10.2 mg, 0.048 mmol) and (AuCl)_2_dppb (21.1 mg, 0.024 mmol) were added to a previously prepared solution of Cs_2_CO_3_ (19.5 mg, 0.06 mmol) in 2-propanol (5 mL). The suspension was maintained under stirring at 50 °C for 2 days. Then, it was cooled to room temperature, and the resulting solid was filtered and recrystallized with dichloromethane/hexane to obtain the pure product in 47% yield (13.1 mg, 0.01 mmol).

^1^H NMR (400 MHz, CDCl_3_): δ 7.94 (ddd, *J* = 7.6, 1.4, 0.7 Hz, 2H_10_), 7.81–7.75 (m, 10H_Ph_), 7.68 (ddd, *J* = 7.0, 5.6, 1.4 Hz, 2H_9_), 7.50 (dt, *J* = 8.2, 0.9 Hz, 2H_6_), 7.46–7.39 (m, 10H_Ph_), 7.38–7.33 (m, 4H_11,7_), 7.30 (dd, *J* = 8.9, 1.1 Hz, 2H_8_), 7.27 (d, *J* = 1.3 Hz, 2H_12_), 2.59 (q, *J* = 8.4, 7.3 Hz, 4H_CH_2__), 2.04 (d, *J* = 19.8 Hz, 4H_CH_2__). ^31^P{^1^H} NMR (161.9 MHz, CDCl_3_, ppm): δ 34.68.

#### Synthesis of **1b**

The synthesis of **1b** was performed following the same procedure as that for **1a** by substitution of (AuCl)_2_dppb for (AuCl)_2_DPEphos (19.4 mg, 0.02 mmol). Yield 58%.

^1^H NMR (400 MHz, CDCl_3_): δ 7.95 (ddd, *J* = 7.5, 1.4, 0.7 Hz, 2H_9_), 7.82 (ddd, *J* = 7.0, 5.6, 1.4 Hz, 2H_10_), 7.78 (ddd, *J* = 7.6, 1.4, 0.6 Hz, 2H_6_), 7.67 (ddd, *J* = 12.0, 8.2, 1.4 Hz, 4H_Ph_), 7.54–7.49 (m, 2H_A_), 7.47–7.39 (m, 10H_Ph_), 7.37–7.31 (m, 6H_Ph_), 7.28–7.24 (m, 2H_12_), 7.09–7.01 (m, 4H_7,8_), 6.98 (tt, *J* = 7.6, 1.4 Hz, 2H_11_), 6.80 (ddd, *J* = 8.3, 7.4, 2.3 Hz, 4H_B,D_), 6.28–6.23 (m, 2H_C_). ^31^P{^1^H} NMR (161.9 MHz, CDCl_3_, ppm): δ 37.69.

#### Synthesis of **1c**

The synthesis of **1c** was performed following the same procedure as that for **1a** by substitution of (AuCl)_2_dppb for (AuCl)_2_Xantphos (25.4 mg, 0.024 mmol). Yield 61%.

^1^H NMR (400 MHz, CDCl_3_): δ 7.89–7.82 (m, 4H_9,10_), 7.64–7.62 (m, 2H_6_), 7.58–7.56 (m, 2H_7_), 7.53–7.38 (m, 6H_8,11,12_), 7.34–7.27 (m, 10H_Ph_), 7.22 (ddd, *J* = 7.6, 7.0, 1.4 Hz, 10H_Ph_), 7.13 (td, *J* = 7.4, 1.0 Hz, 2H_D_), 6.97 (td, *J* = 7.8, 1.2 Hz, 2H_A_), 6.42 (ddd, *J* = 10.8, 7.7, 1.6 Hz, 1H_B_), 1.71 (s, 6H_CH_3__). ^31^P{^1^H} NMR (161.9 MHz, CDCl_3_, ppm): δ 34.68.

#### Synthesis of **1d**

The synthesis of **1d** was performed following the same procedure as that for **1a** by substitution of (AuCl)_2_dppb for (AuCl)_2_BiPheP (21 mg, 0.02 mmol). Yield 43%.

^1^H NMR (400 MHz, CDCl_3_): δ 7.87 (ddd, *J* = 7.6, 1.5, 0.7 Hz, 2H_D_), 7.74–7.67 (m, 10H_Ph_), 7.61 (ddd, *J* = 7.0, 5.6, 1.4 Hz, 2H_9_), 7.46–7.41 (m, 4H_10,6_), 7.39–7.32 (m, 12H_Ph,7_), 7.32–7.26 (m, 4H_B,A_), 7.26–7.20 (m, 8H_8,12,11,C_). ^31^P{^1^H} NMR (161.9 MHz, CDCl_3_, ppm): δ 38.61.

#### Synthesis of **2a**

The synthesis of **2a** was performed following the same procedure as that for **1a** by substitution of dibenzofuran-4-boronic acid for 9,9-dimethyl-9H-fluoren-2-ylboronic acid (10.5 mg, 0.044 mmol). Yield 51%.

^1^H NMR (400 MHz, CDCl_3_): δ 7.69–7.58 (m, 14H_6,9,10,Ph_), 7.50–7.44 (m, 2H_7_), 7.41–7.36 (m, 10H_Ph_), 7.35–7.32 (m, 2H_8_), 7.23 (td, *J* = 7.4, 1.3 Hz, 2H_13_), 7.17 (dd, *J* = 7.3, 1.3 Hz, 2H_11_), 2.40 (m, 4H_CH_2__), 1.88–1.82 (m, 4H_CH_2__), 1.43 (s, 12H_CH_3__). ^31^P{^1^H} NMR (161.9 MHz, CDCl_3_, ppm): δ 38.51.

#### Synthesis of **2b**

The synthesis of **2b** was performed following the same procedure as that for **1b** by substitution of dibenzofuran-4-boronic acid for 9,9-dimethyl-9H-fluoren-2-ylboronic acid (19.05 mg, 0.08 mmol). Yield 60%.

^1^H NMR (400 MHz, CDCl_3_): δ 7.58–7.55 (m, 2H_9_), 7.51–7.44 (m, 6H_10,6,13_), 7.42–7.40 (m, 2H_11_), 7.38–7.33 (m, 4H_7,8_), 7.31–7.29 (m, 2H_A_), 7.24–7.21 (m, 5H_Ph_), 7.18–7.08 (m, 15H_Ph_), 6.98 (td, *J* = 7.7, 2.5 Hz, 4H_B,D_), 6.77–6.71 (m, 2H_C_), 1.36 (s, 3H_CH_3__), 1.31 (s, 3H_CH_3__). ^31^P{^1^H} NMR (161.9 MHz, CDCl_3_, ppm): δ 36.06.

#### Synthesis of **2c**

The synthesis of **2c** was performed following the same procedure as that for **1c** by substitution of dibenzofuran-4-boronic acid for 9,9-dimethyl-9*H*-fluoren-2-ylboronic acid (11.4 mg, 0.048 mmol). Yield 56%.

^1^H NMR (400 MHz, CDCl_3_): δ 7.83 (d, *J* = 5.7 Hz, 2H_9_), 7.71–7.68 (m, 2H_10_), 7.64 (d, *J* = 7.5 Hz, 2H_6_), 7.59 (dd, *J* = 7.8, 1.5 Hz, 2H_13_), 7.57–7.51 (m, 2H_A_), 7.37–7.16 (m, 20H_Ph_), 7.15–7.05 (m, 8H_8,B,7,11_), 6.49 (ddd, *J* = 10.5, 7.7, 1.5 Hz, 2H_C_). ^31^P{^1^H} NMR (161.9 MHz, CDCl_3_, ppm): δ 35.14.

#### Synthesis of **2d**

The synthesis of **2d** was performed following the same procedure as that for **1d** by substitution of dibenzofuran-4-boronic acid for 9,9-dimethyl-9*H*-fluoren-2-ylboronic acid (23.8 mg, 0.1 mmol). Yield 54%.

^1^H NMR (400 MHz, CDCl_3_): δ 7.76–7.72 (m, 4H_D,9_), 7.63–7.55 (m, 6H_10,A,B_), 7.53–7.53–7.27 (m, 20H_Ph_), 7.15–7.09 (m, 2H_6_), 7.04–6.99 (m, 2H_13_), 6.93 (tt, *J* = 7.6, 1.4 Hz, 2H_C_), 6.78 (ddd, *J* = 8.2, 7.4, 2.3 Hz, 4H_7,8_), 6.24 (ddd, *J* = 7.7, 4.5, 1.2 Hz, 2H_11_), 1.57 (s, 6H_CH_3__), 1.45 (s, 6H_CH_3__). ^31^P{^1^H} NMR (161.9 MHz, CDCl_3_, ppm): δ 37.97.

## Supporting Information Available

Structural characterization; absorption spectra at different concentrations; absorption vs concentration plot; HOMO and LUMO molecular orbitals; calculated absorption spectra in DCM; TD-DFT plots of the main electronic states; tables with the calculated *S_n_* and *T_n_* energies by TD-DFT with their contribution; tables with the crystal data (PDF)
