# Supplementary material for: Intra- vs Intermolecular Aurophilic Contacts in Dinuclear Gold(I) Compounds: Impact on the Population of the Triplet Excited State
Source: Inorg Chem. 2022 Dec 13;61(51):20931–41. doi: 10.1021/acs.inorgchem.2c03351 (PMC9795547; doi:10.1021/acs.inorgchem.2c03351)
Supplement: Supplementary file 1 — ic2c03351_si_001.pdf [file ic2c03351_si_001.pdf]

Intra vs intermolecular aurophilic contacts in dinuclear gold(I) compounds. Impact in the population of the triplet excited state.

Araceli de Aquino,[a,b] Jas S. Ward,[c] Kari Rissanen,[c] Gabriel Aullón,[a,d] João Carlos Lima,[e,*] Laura Rodríguez[a,b,*]

[a] *Departament de Química Inorgànica i Orgànica. Secció de Química Inorgànica. Universitat de Barcelona, Martí i Franquès 1-11, 08028 Barcelona, (Spain). e-mail: laura.rodriguez@qi.ub.es*

[b] *Institut de Nanociència i Nanotecnologia (IN2UB). Universitat de Barcelona, 08028 Barcelona (Spain)*

[c] *Department of Chemistry, Nanoscience Center, University of Jyvaskyla, 40014 Jyvaskyla (Finland)*

[d] *Institut de Química Teòrica i Computacional (IQTCUB). Universitat de Barcelona, 08028 Barcelona, (Spain)*

[e] *LAQV-REQUIMTE, Departamento de Química, Faculdade de Ciências e Tecnologia, Universidade Nova de Lisboa, 2829-516 Caparica, Portugal. e-mail: lima@fct.unl.pt*

Supporting Information

Associated Content

pages

Structural characterization (including ^{1}H, ^{31}P NMR and MALDI-TOF MS(+)) ………….………… S3-S14

Absorption spectra at different concentrations ………………………………………………………. S15

Absorption vs Concentration plot ………………………………………………………………… S16

HOMO and LUMO molecular orbitals …………………………………………………………….. S17-S20

Calculated absorption spectra in DCM ……………………………………………………………... S21

TD-DFT plots of the main electronic states ……………………………………………………….. S22-S23

Tables with the calculated S_n and T_n energies by TD-DFT with their contribution …………………. S24-S26

Tables with the crystal data ………………………………………………………………………... S27-S28

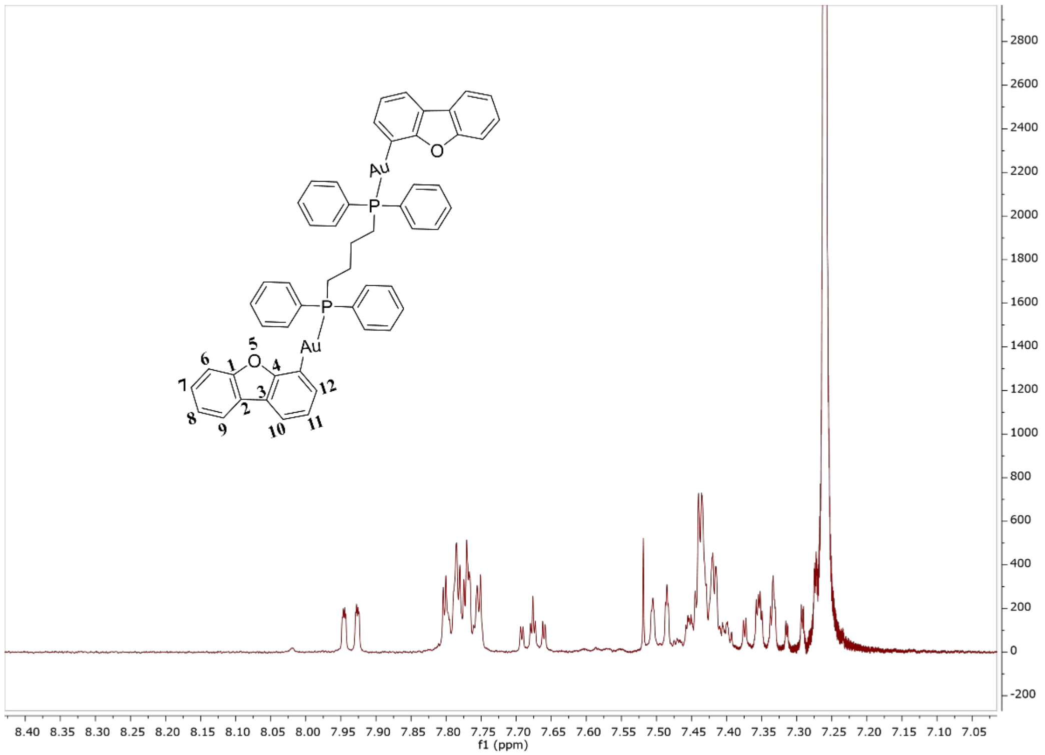

Figure S1. ^{1}H NMR spectrum of **1a** in $CDCl_3$.

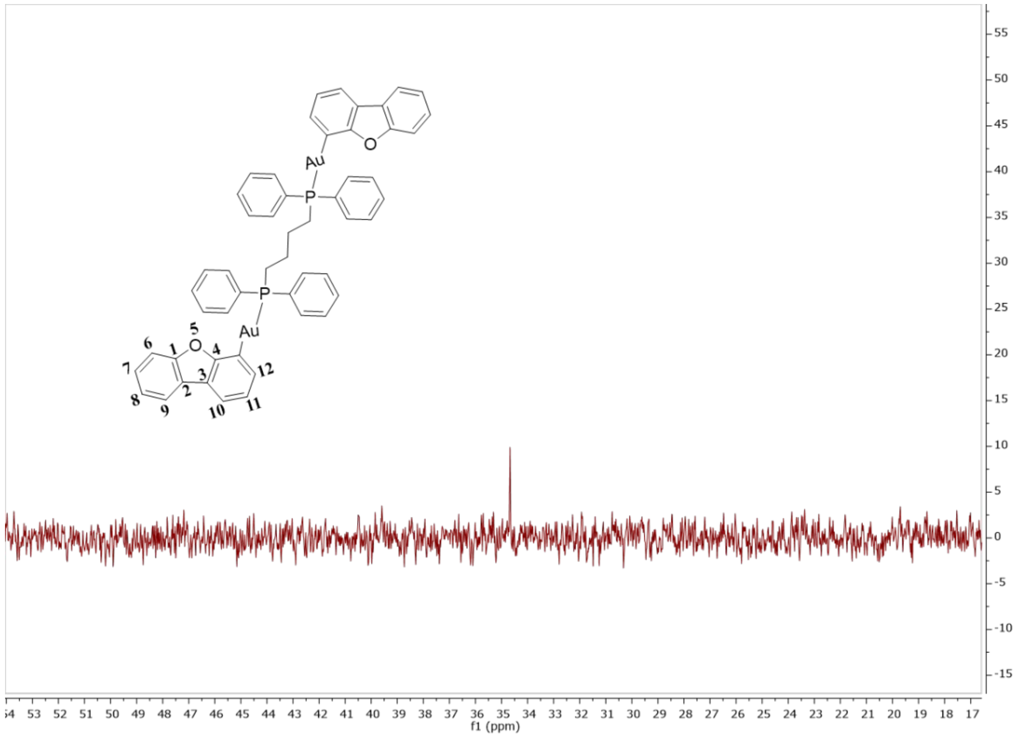

Figure S2. ^{31}P NMR spectrum of **1a** in $CDCl_3$.

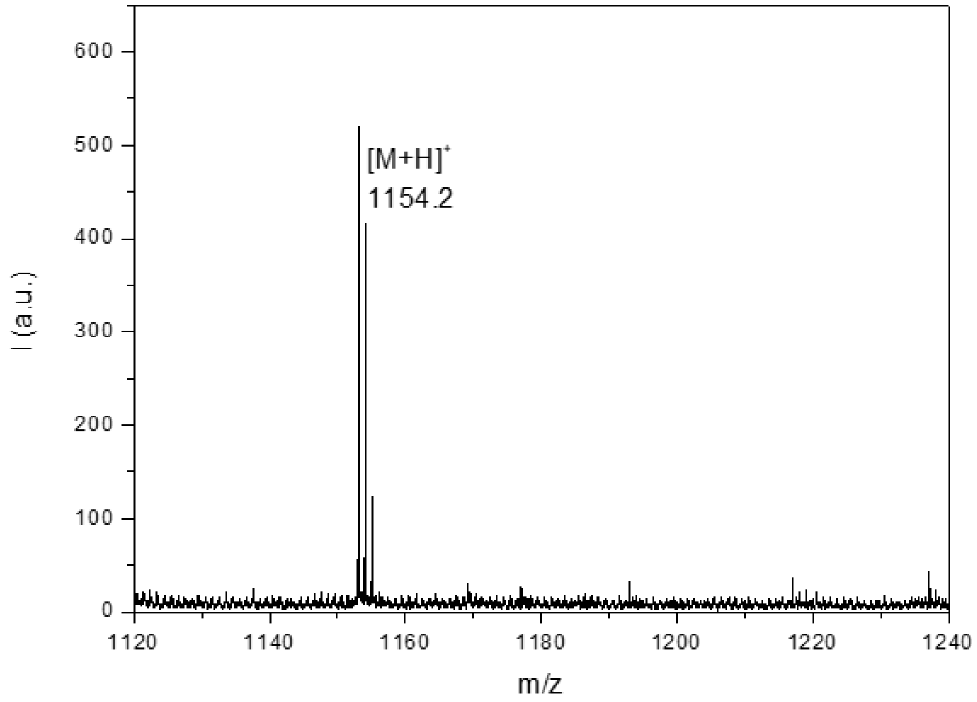

Figure S3. MALDI-TOF MS(+) spectrum of **1a**.

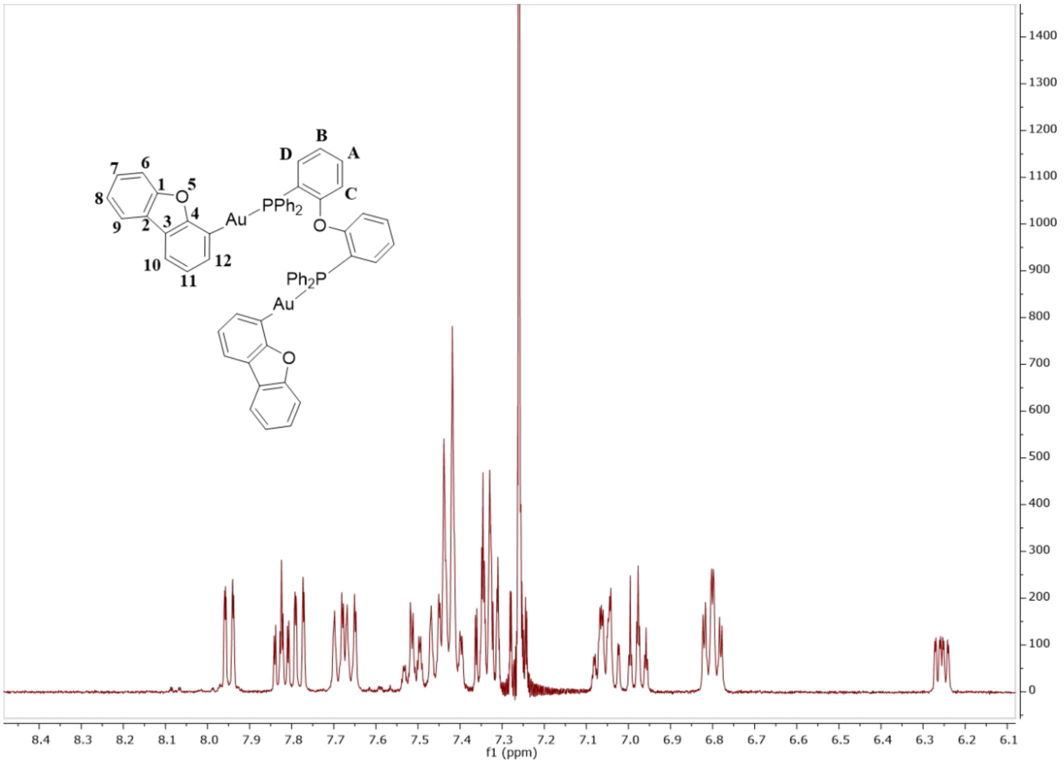

Figure S4. ^{1}H NMR spectrum of **1b** in $CDCl_3$.

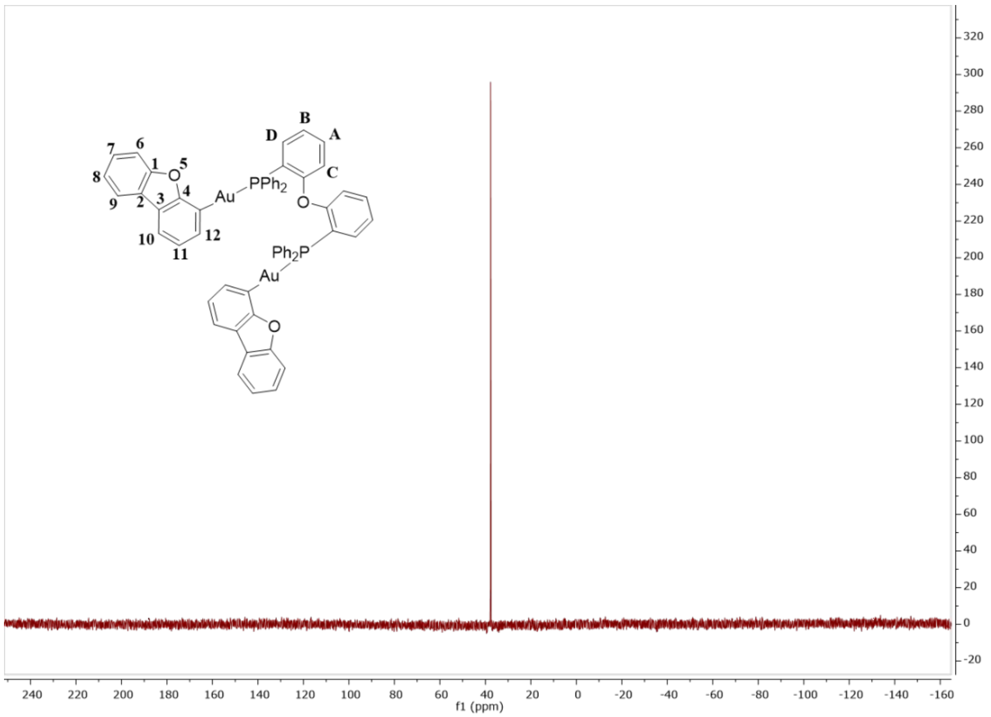

Figure S5. ^{31}P NMR spectrum of **1b** in $CDCl_3$.

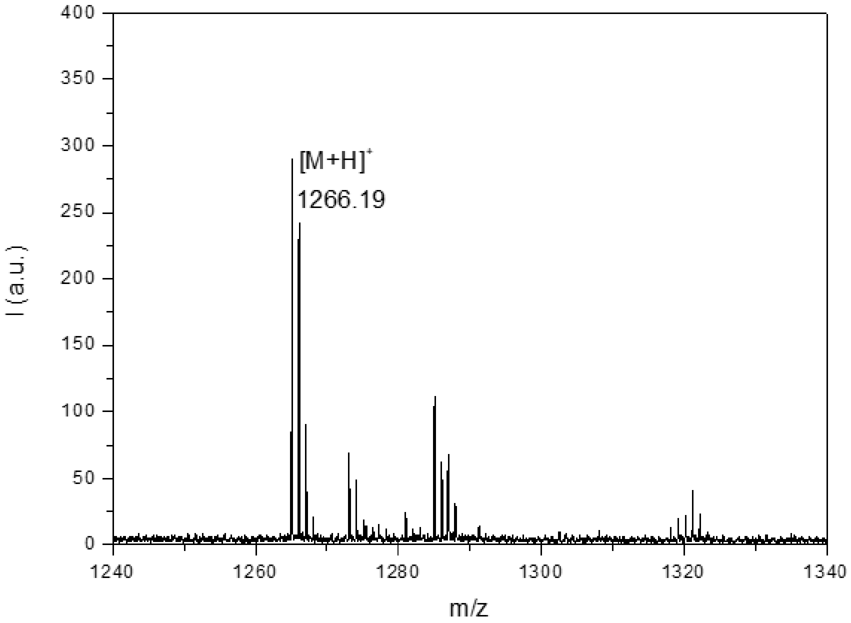

Figure S6. MALDI-TOF MS(+) spectrum of **1b**.

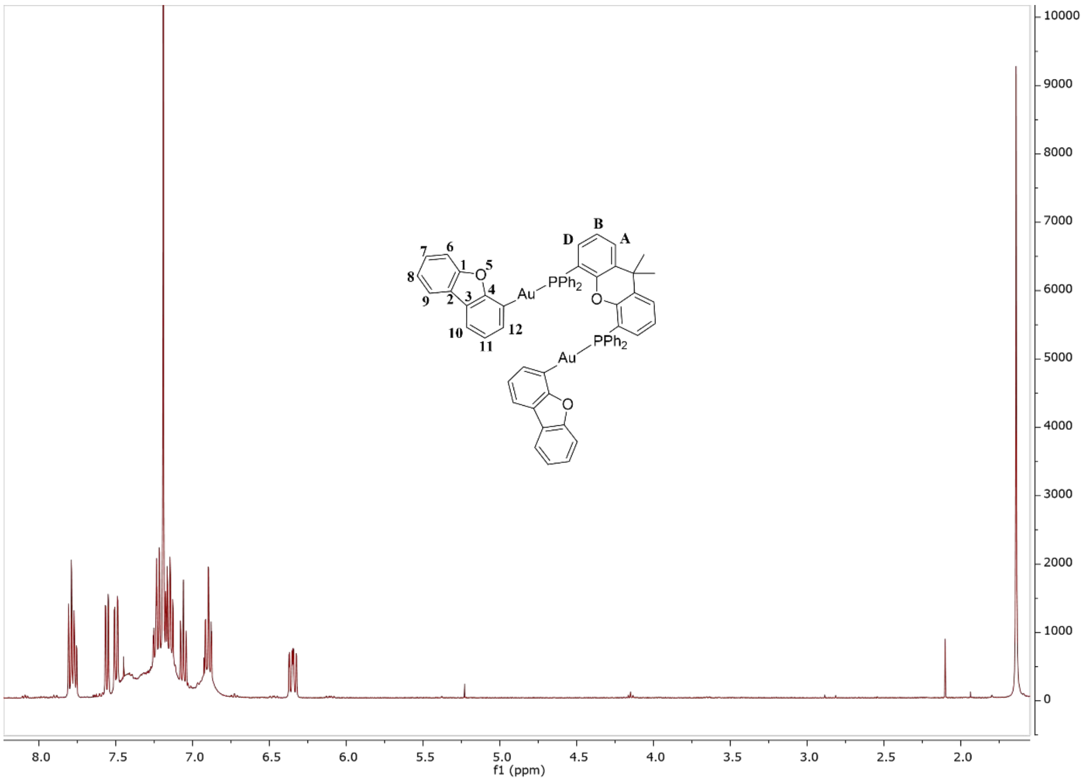

Figure S7. ^{1}H NMR spectrum of **1c** in $CDCl_3$.

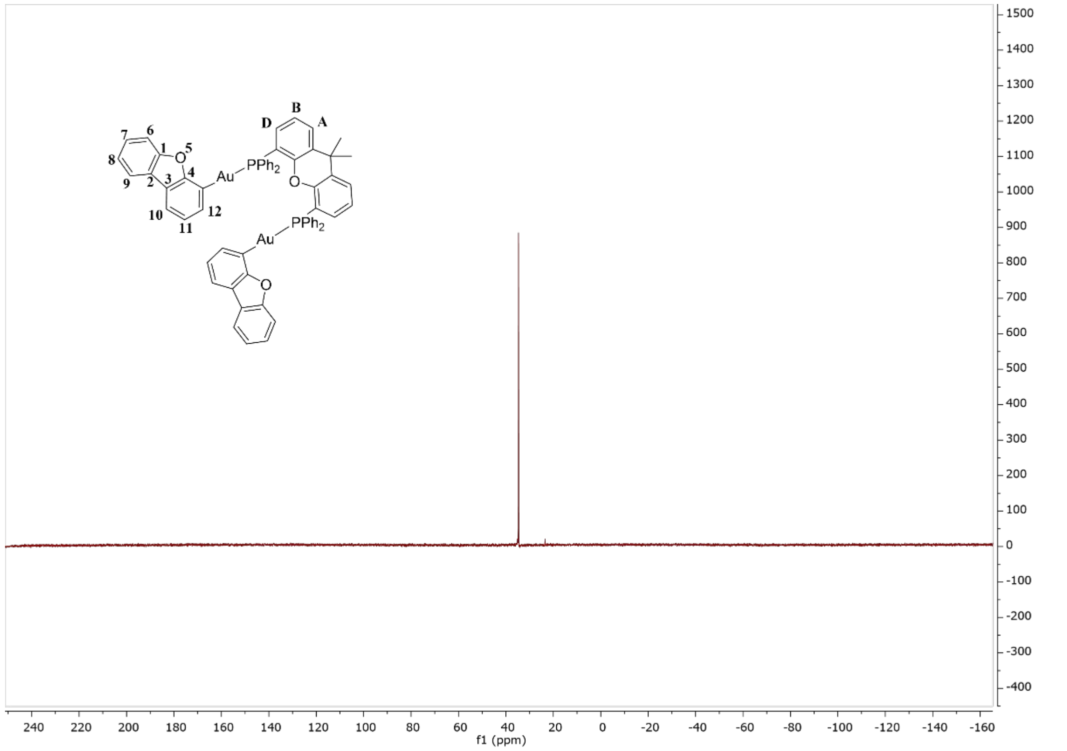

Figure S8. ^{31}P NMR spectrum of **1c** in $CDCl_3$.

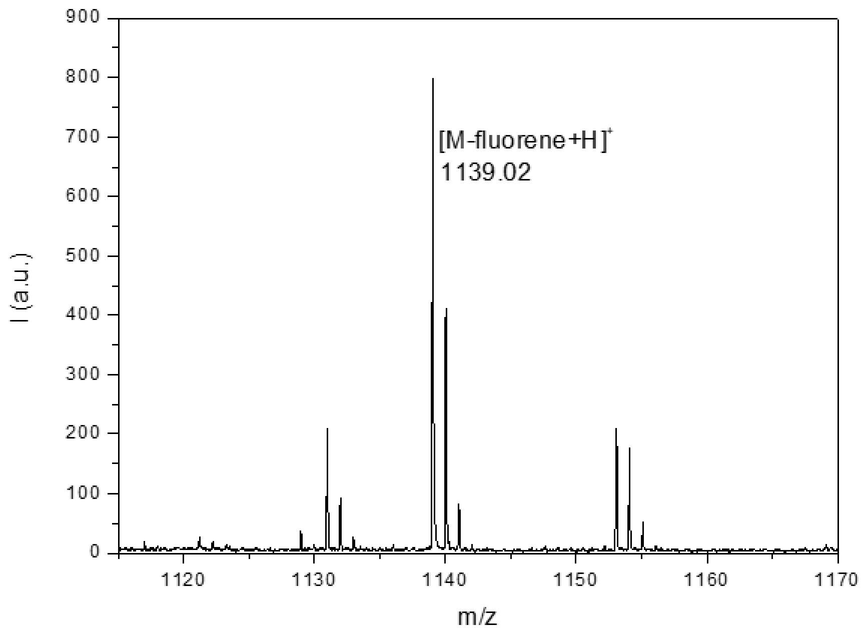

Figure S9. MALDI-TOF MS(+) spectrum of **1c**.

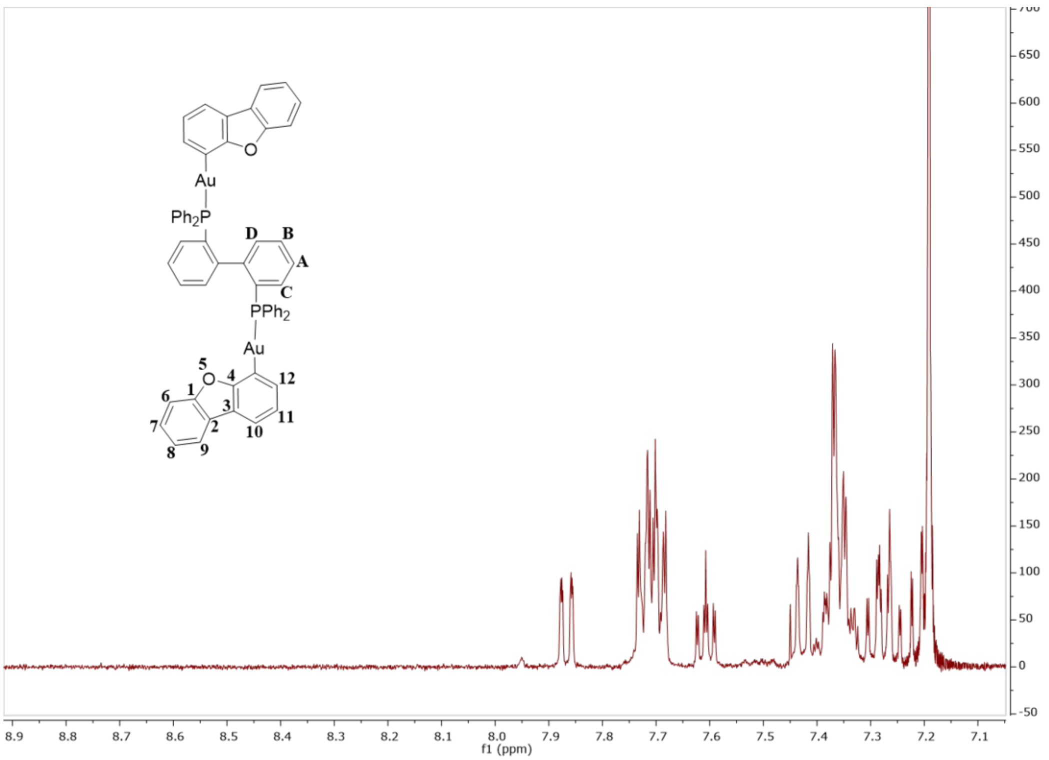

Figure S10. ^{1}H NMR spectrum of **1d** in $CDCl_3$.

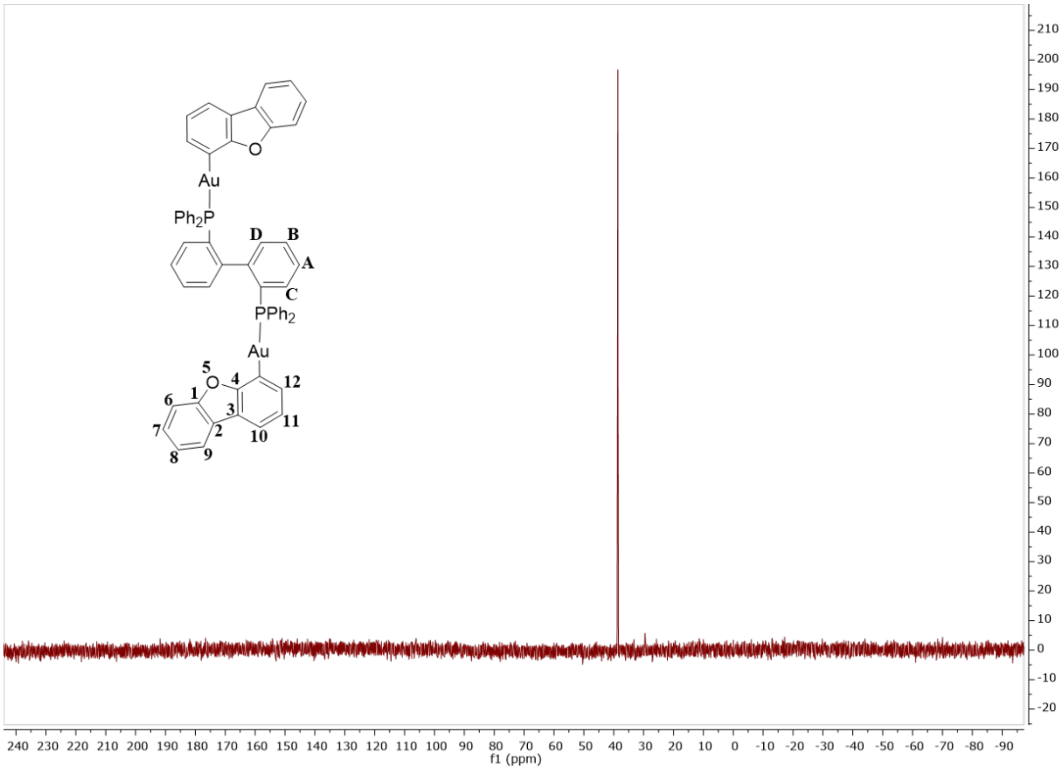

Figure S11. ^{31}P NMR spectrum of **1d** in $CDCl_3$.

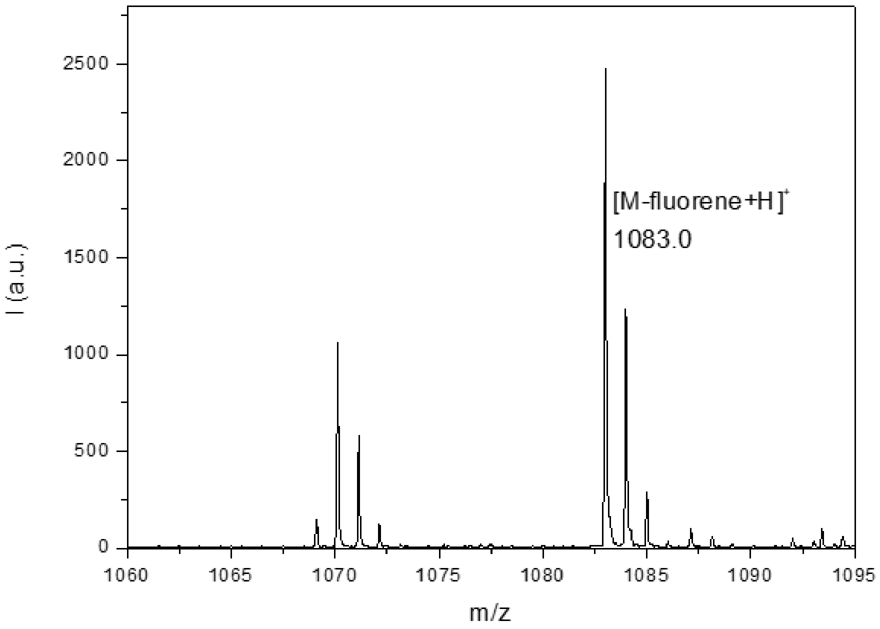

Figure S12. MALDI-TOF MS(+) spectrum of **1d**.

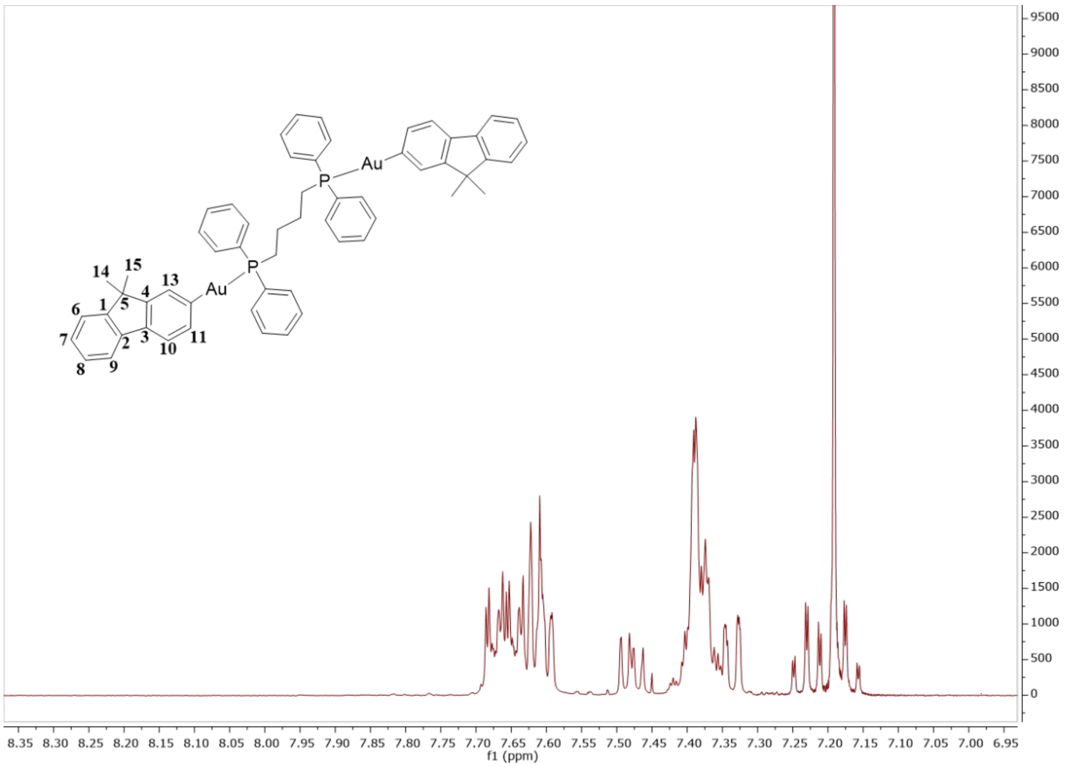

Figure S13. ^{1}H NMR spectrum of **2a** in $CDCl_3$.

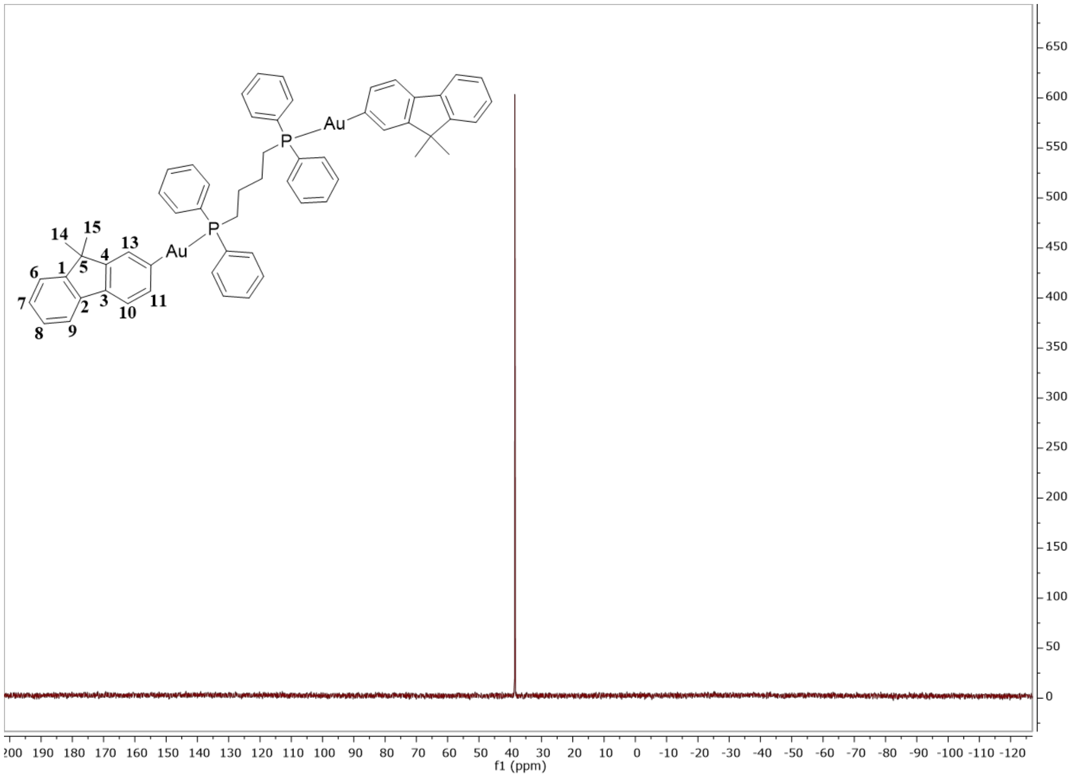

Figure S14. ^{31}P NMR spectrum of **2a** in $CDCl_3$.

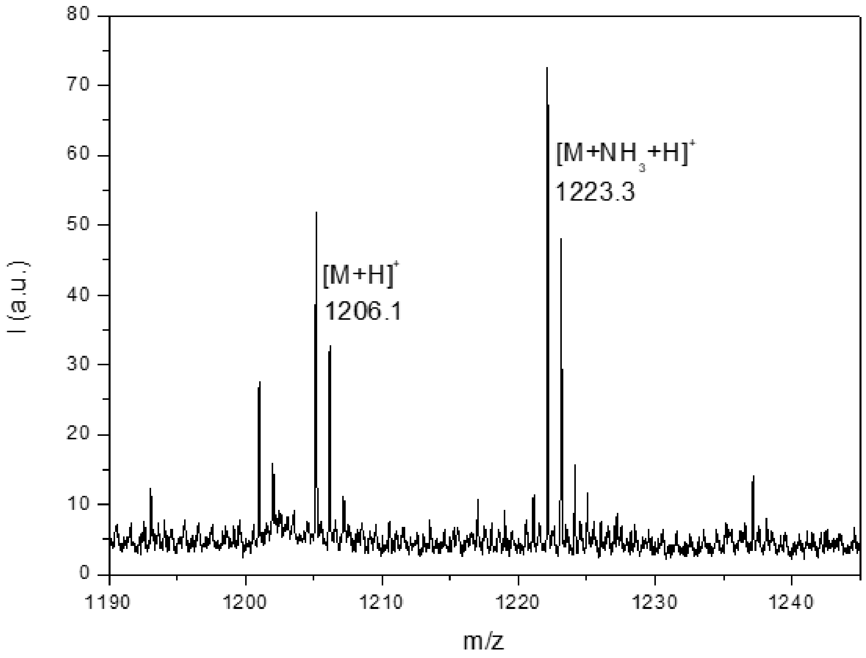

Figure S15. MALDI-TOF MS(+) spectrum of **2a**.

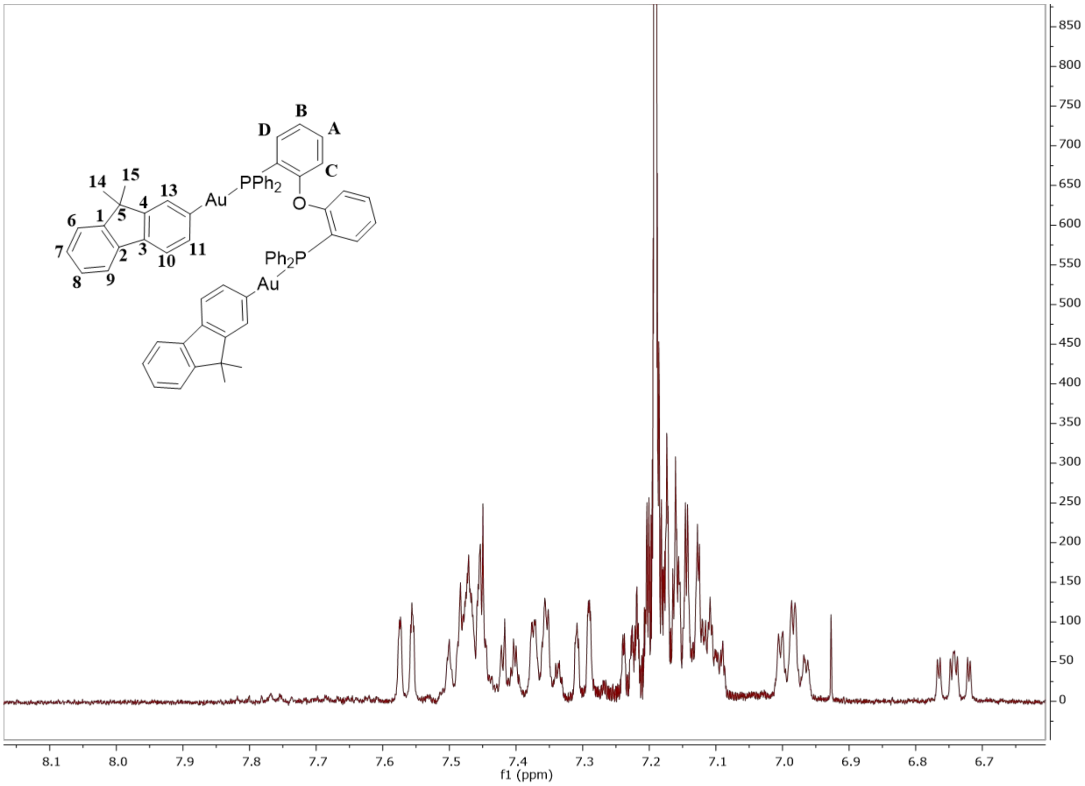

Figure S16. ^{1}H NMR spectrum of **2b** in $CDCl_3$.

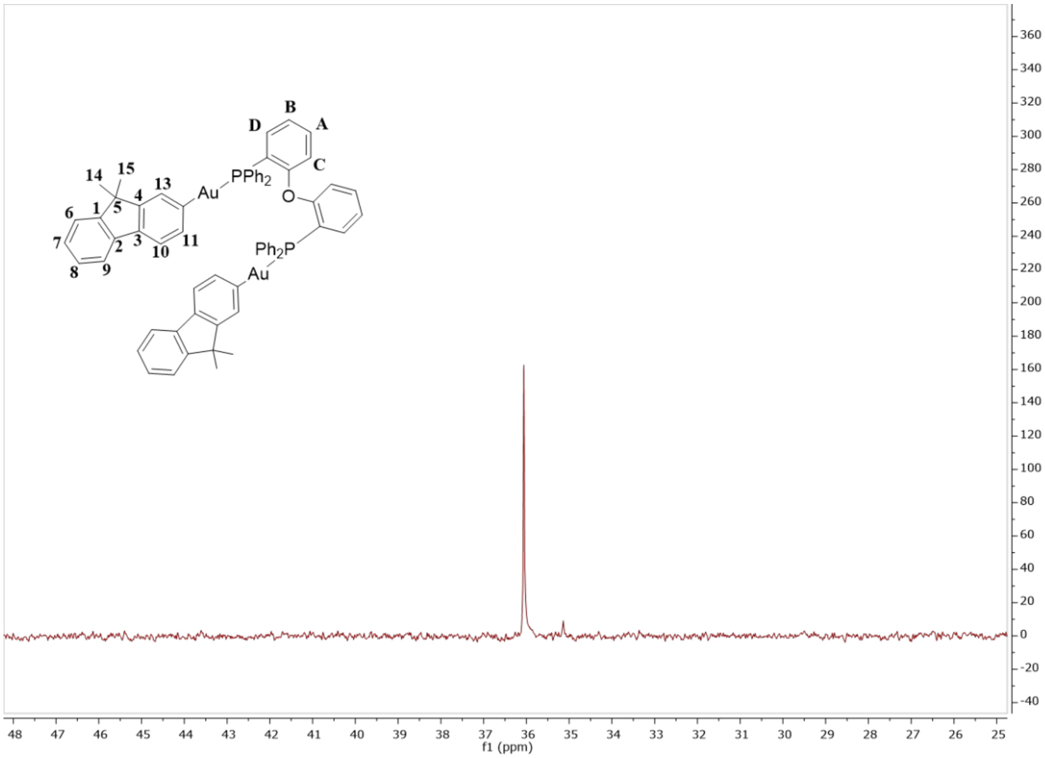

Figure S17. ^{31}P NMR spectrum of **2b** in $CDCl_3$.

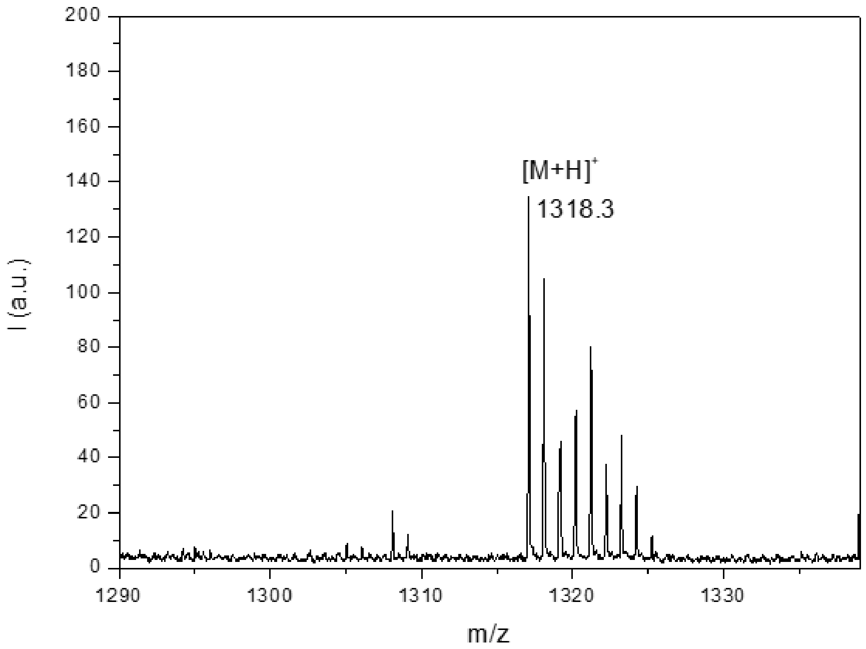

Figure S18. MALDI-TOF MS(+) spectrum of **2b**.

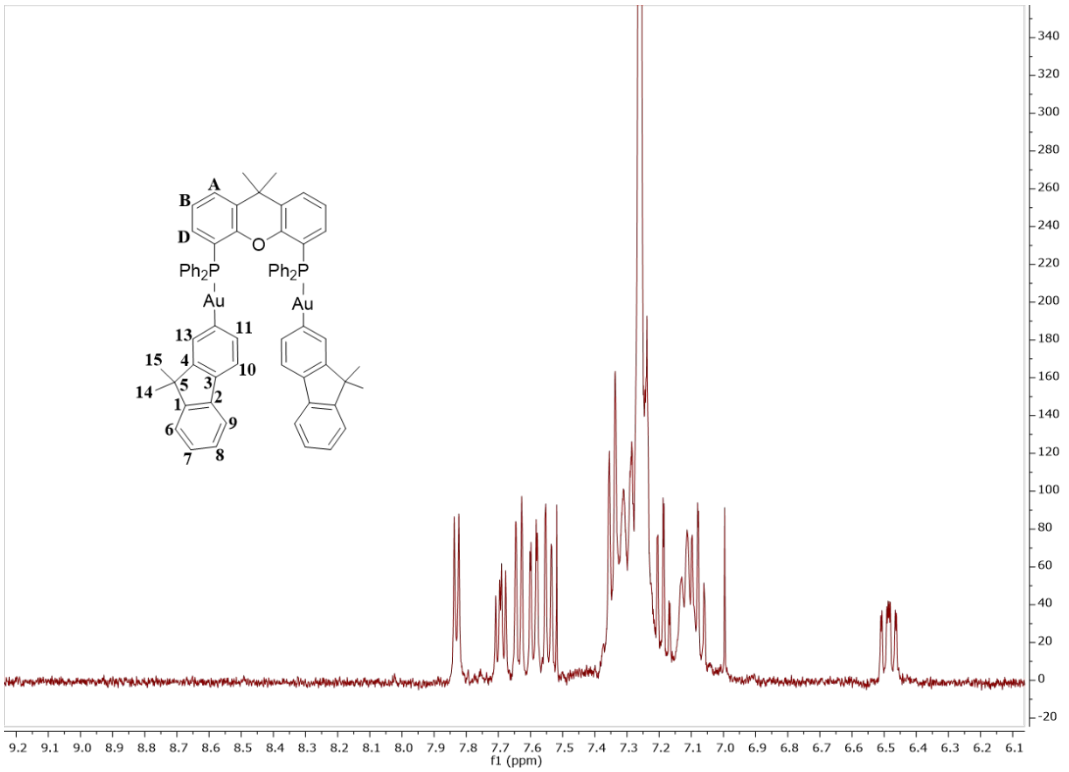

Figure S19. ^{1}H NMR spectrum of **2c** in $CDCl_3$.

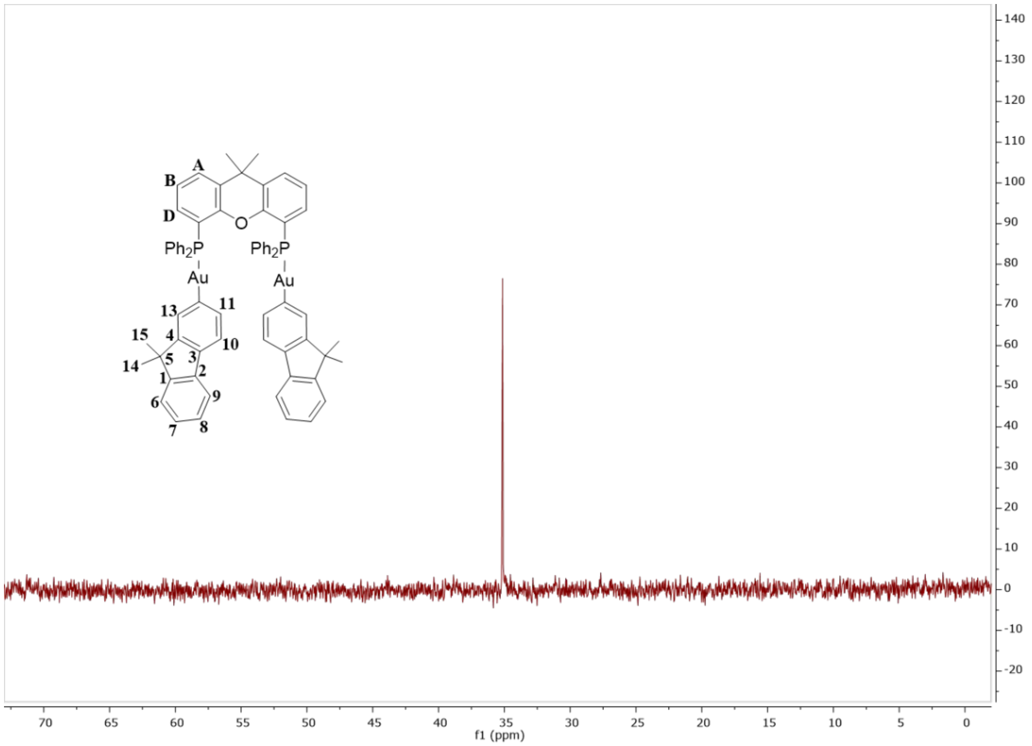

Figure S20. ^{31}P NMR spectrum of **2c** in $CDCl_3$.

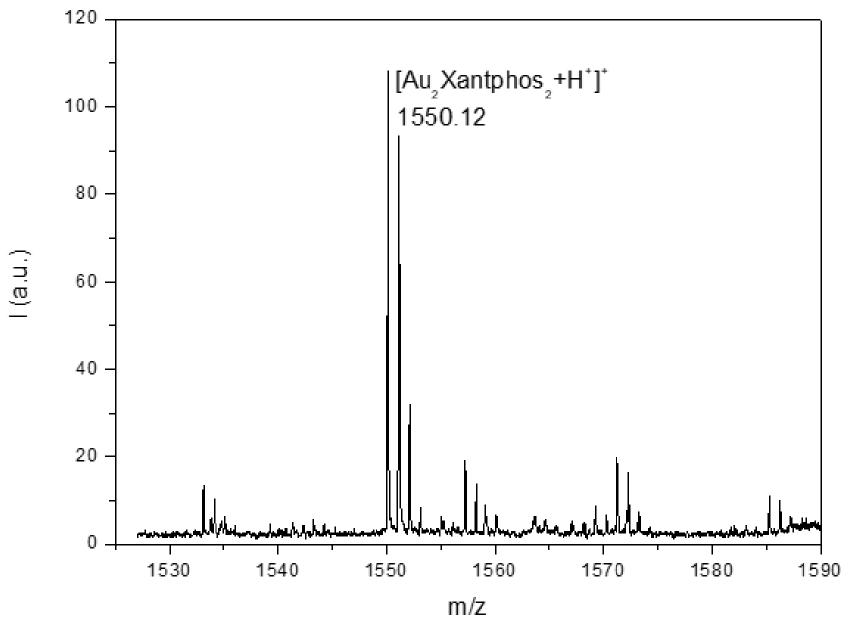

Figure S21. MALDI-TOF MS(+) spectrum of **2c**.

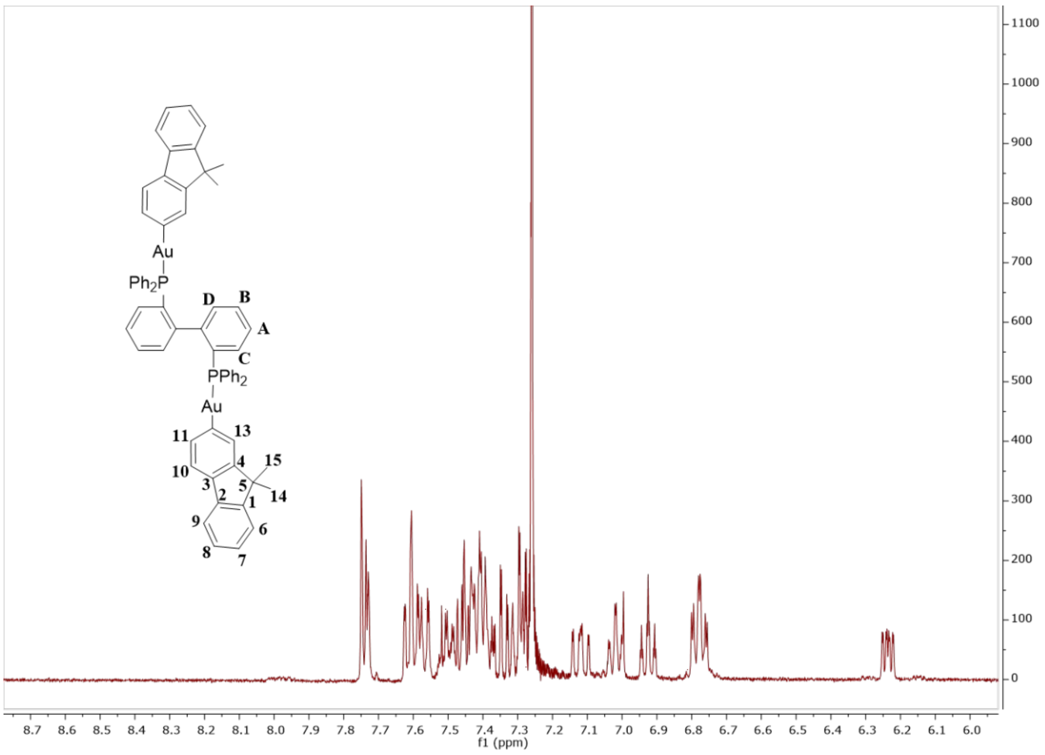

Figure S22. ^{1}H NMR spectrum of **2d** in $CDCl_3$.

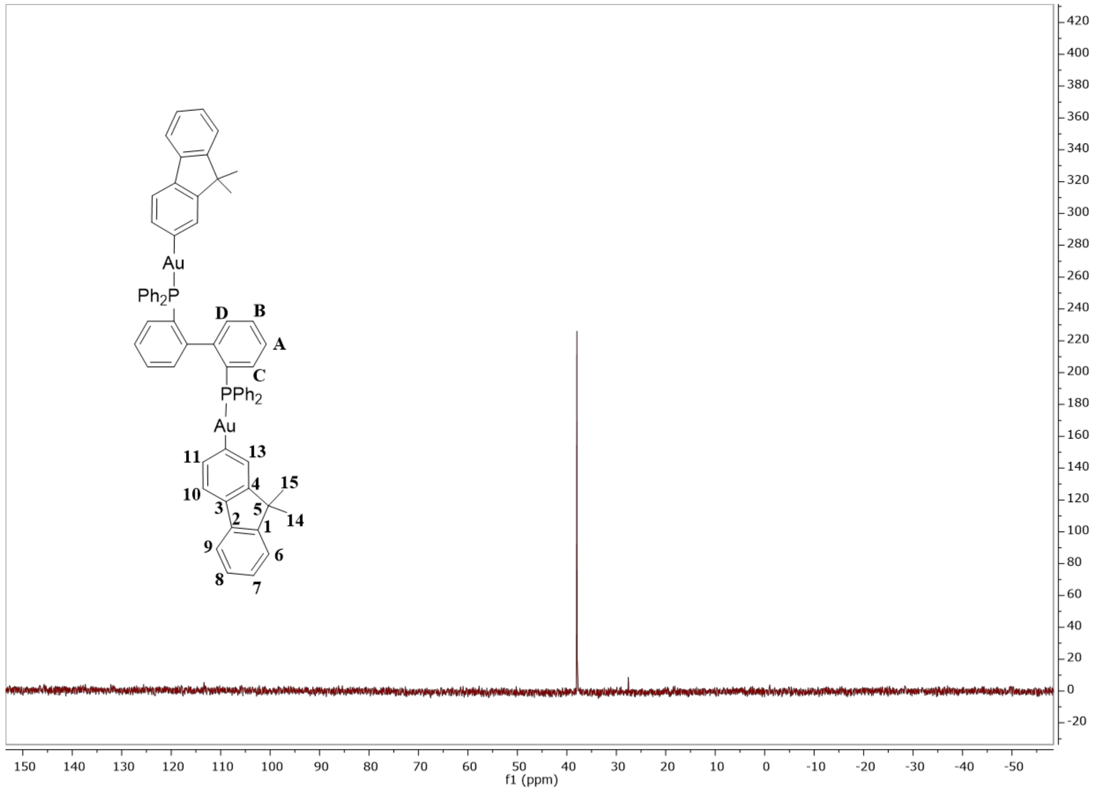

Figure S23. ^{31}P NMR spectrum of **2d** in $CDCl_3$.

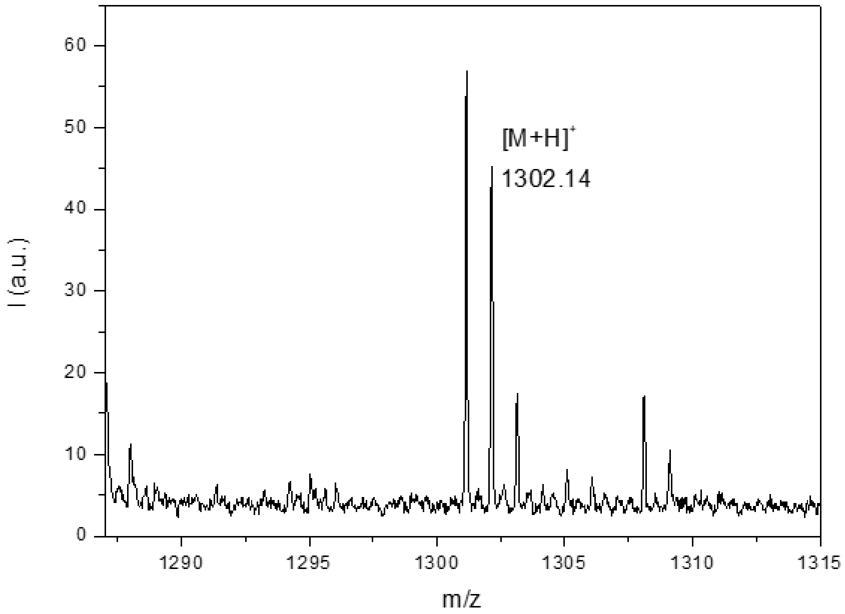

Figure S24. MALDI-TOF MS(+) spectrum of **2d**.

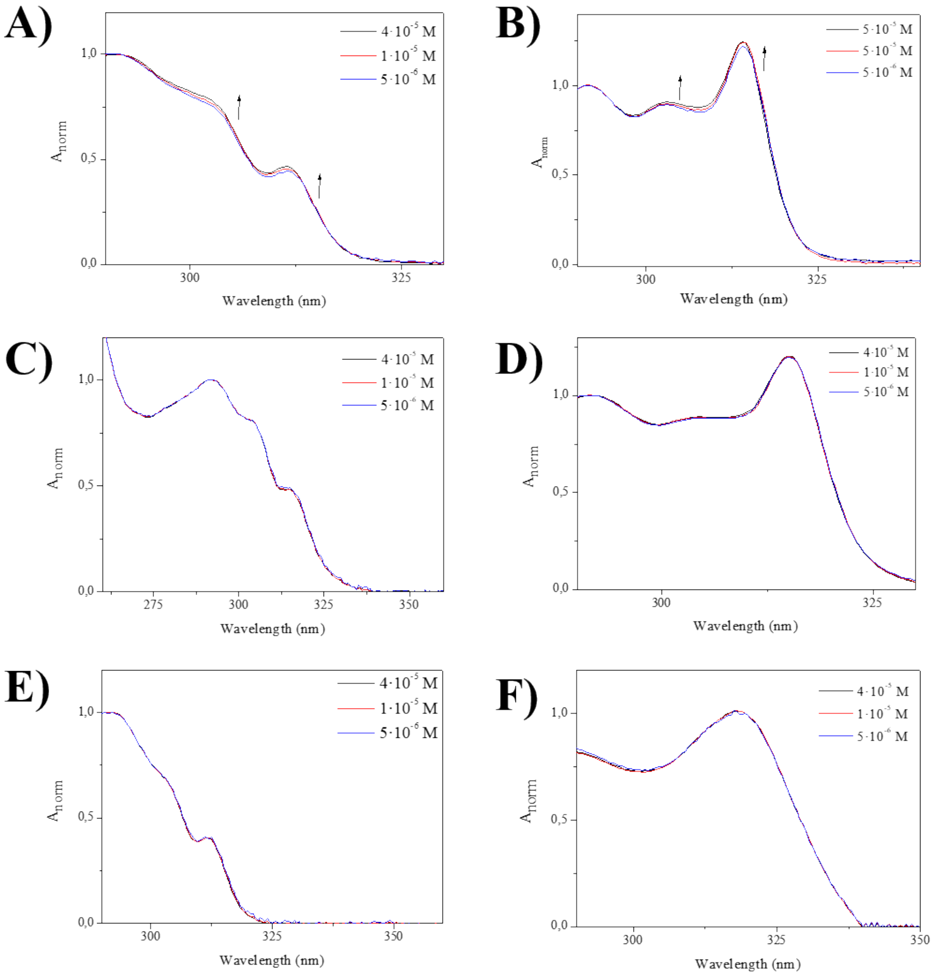

Figure S25. Absorption spectra of **1a** (A), **2a** (B), **1b** (C), **2b** (D), **1d** (E) and **2d** (F) at different concentrations.

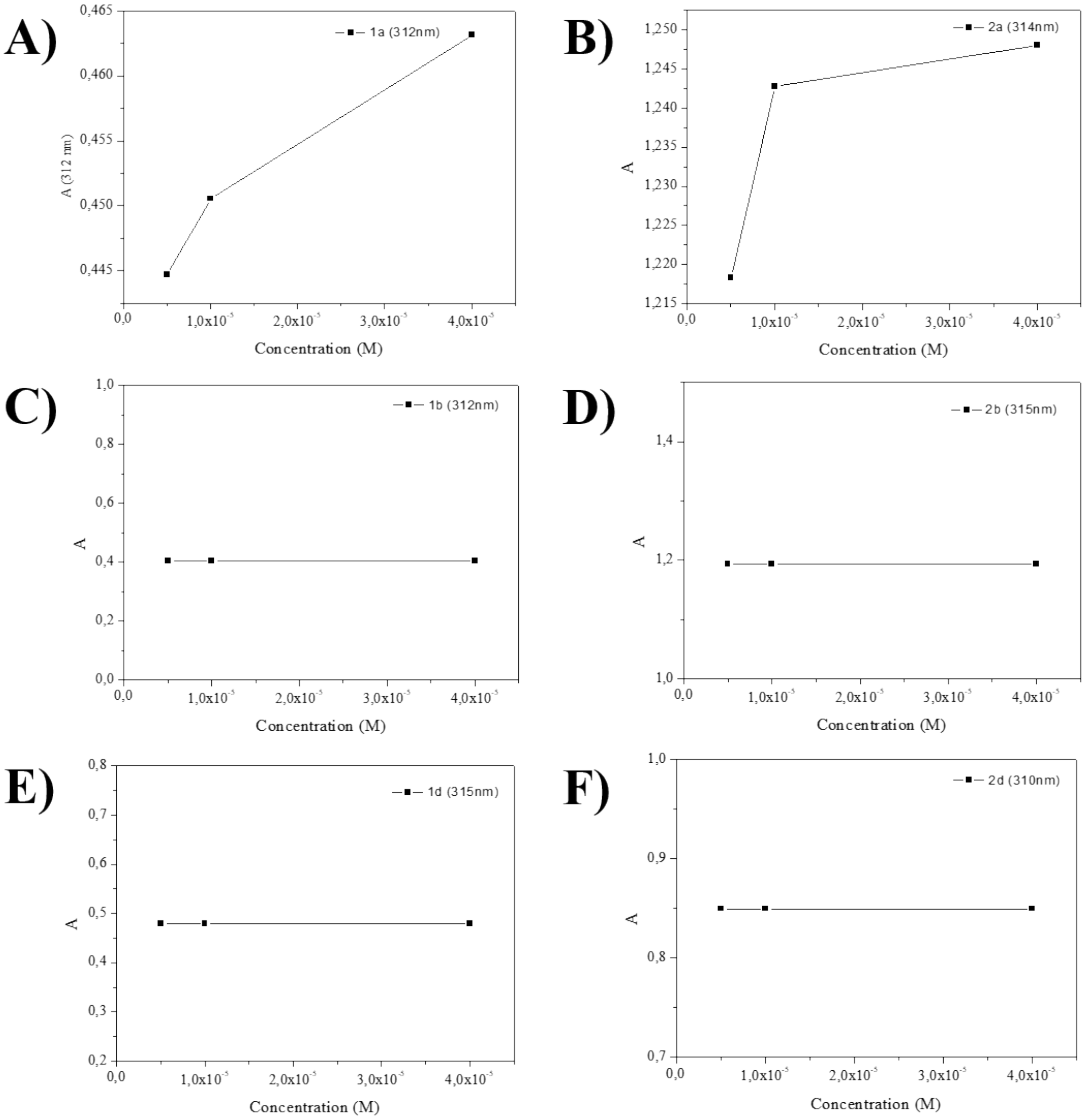

Figure S26. Plot of A280nm/A320nm vs versus Concentration values variation for each compound studied in the previous spectra identifying the presence of aurophilic intermolecular contacts for **1a** (A) and **2a** (B) and the maintenance of intramolecular aurophilic contacts in **1c** (C), **2c** (D) and **1d** (E), **2d** (F).

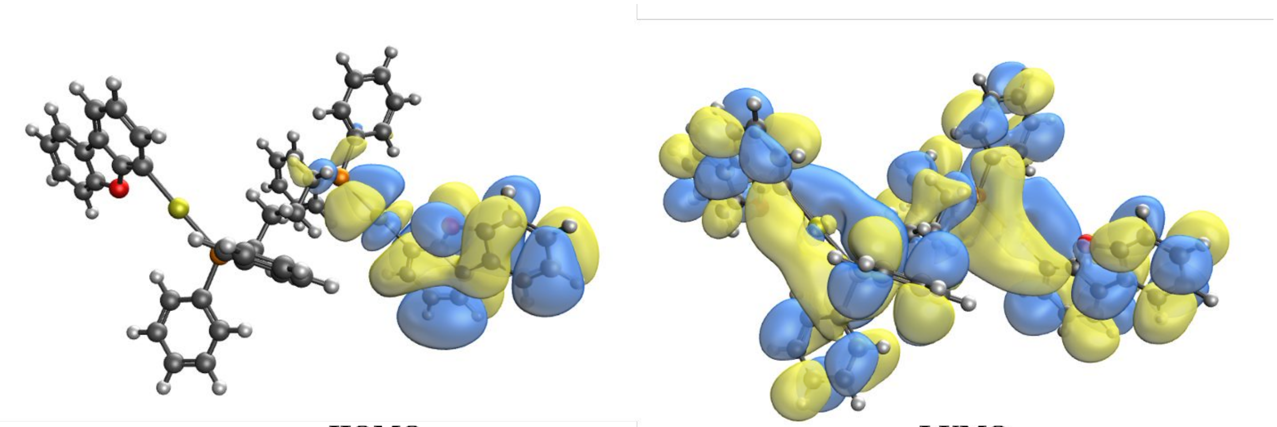

HOMO **LUMO**

Figure S27. HOMO and LUMO orbitals involved in the studied transitions of compound **1a**.

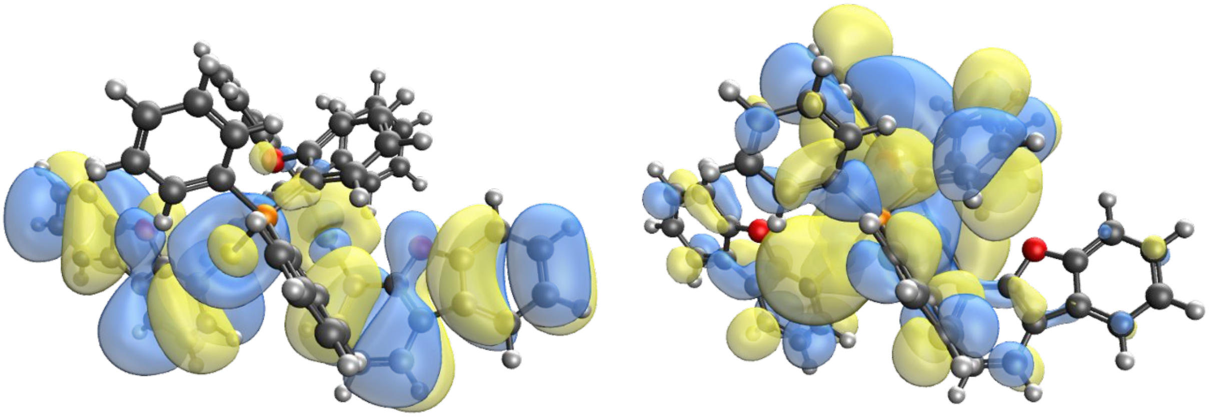

HOMO **LUMO**

Figure S28. HOMO and LUMO orbitals involved in the studied transitions of compound **1b**.

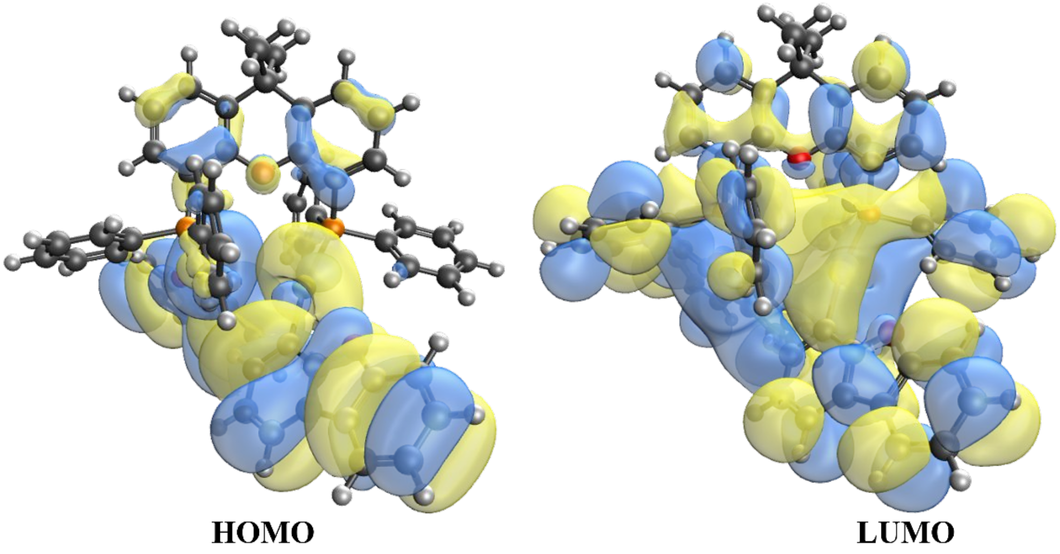

Figure S29. HOMO and LUMO orbitals involved in the studied transitions of compound **1c**.

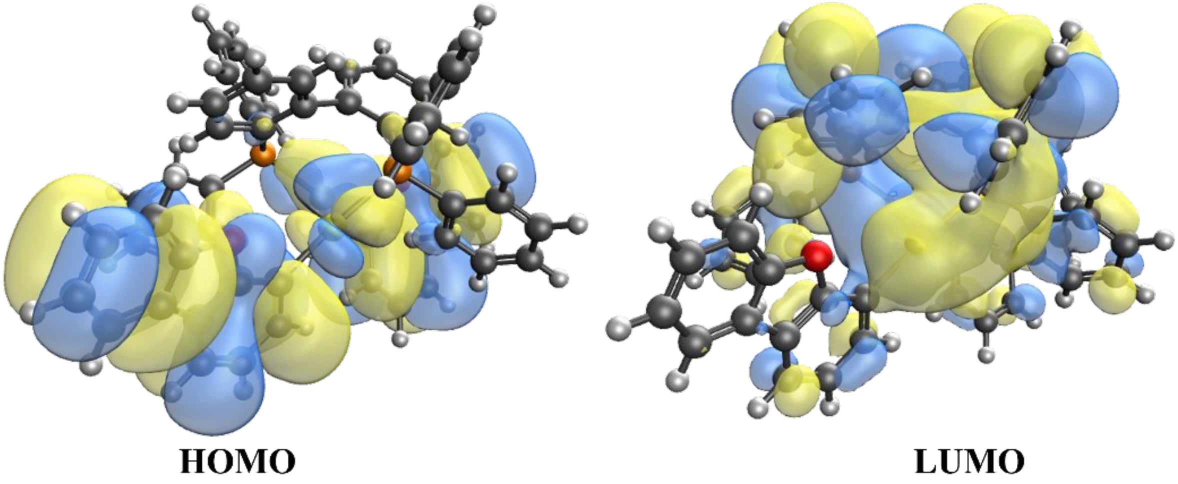

Figure S30. HOMO and LUMO orbitals involved in the studied transitions of compound **1d**.

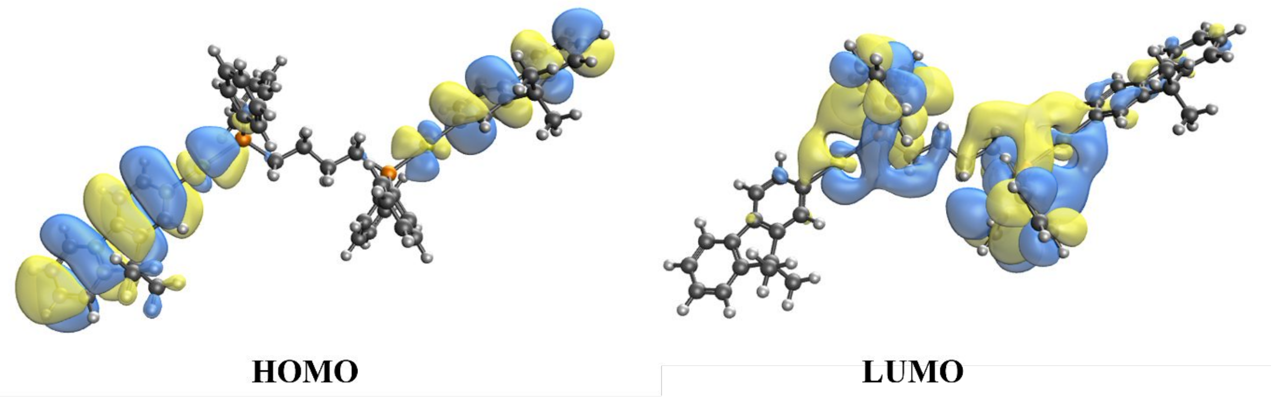

Figure S31. HOMO and LUMO orbitals involved in the studied transitions of compound **2a**.

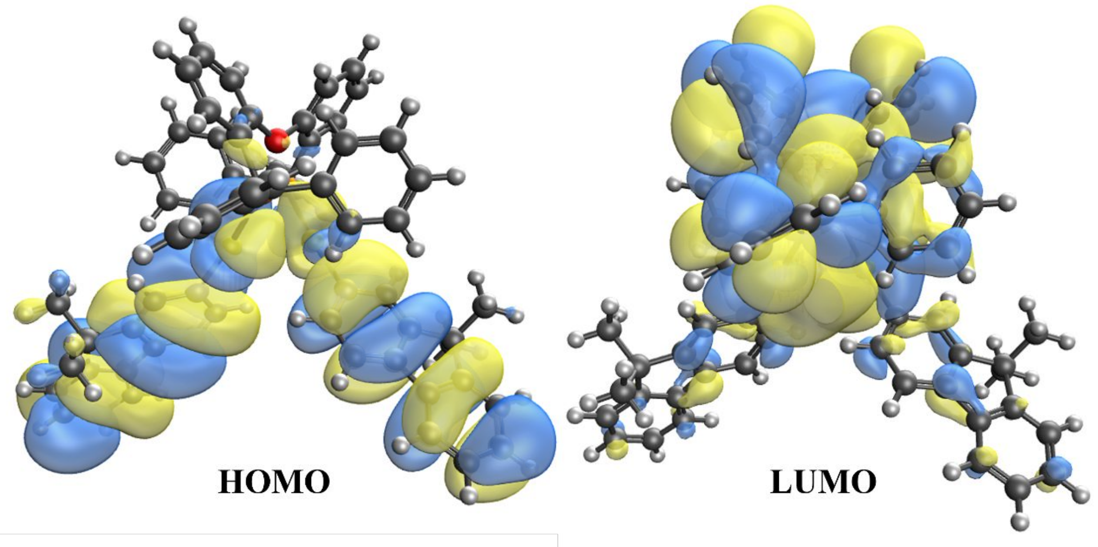

Figure S32. HOMO and LUMO orbitals involved in the studied transitions of compound **2b**.

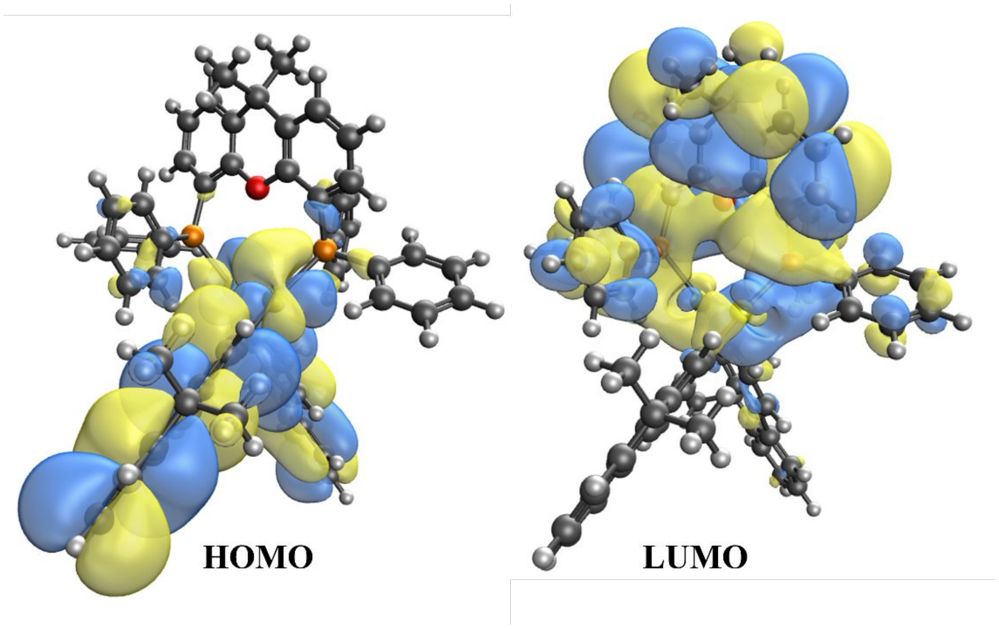

Figure S33. HOMO and LUMO orbitals involved in the studied transitions of compound **2c**.

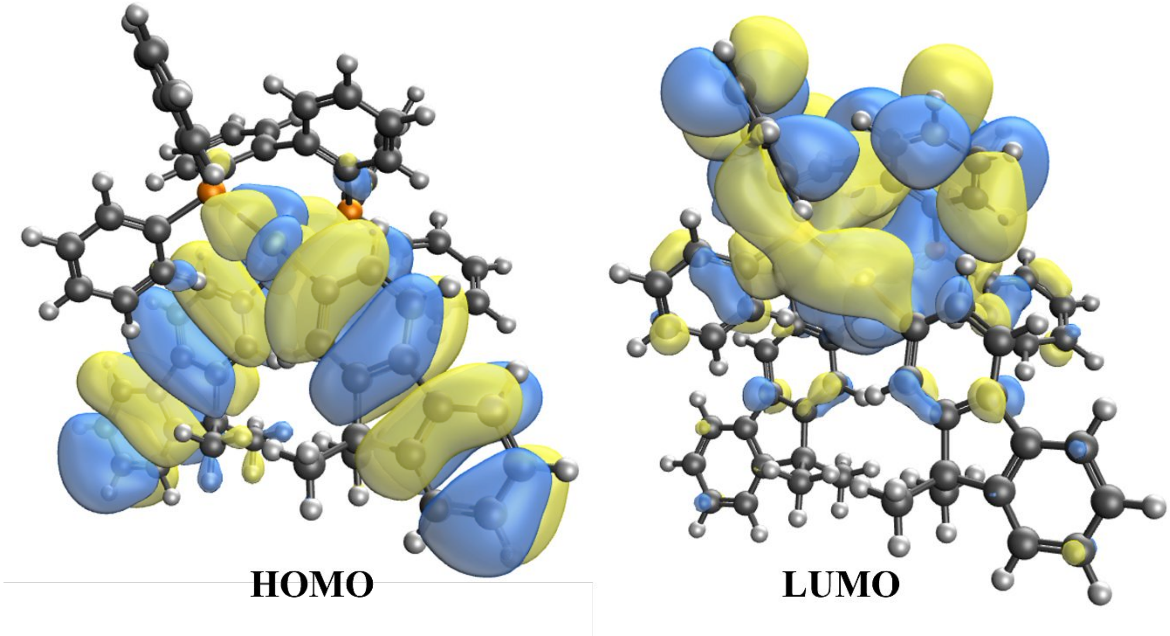

Figure S34. HOMO and LUMO orbitals involved in the studied transitions of compound **2d**.

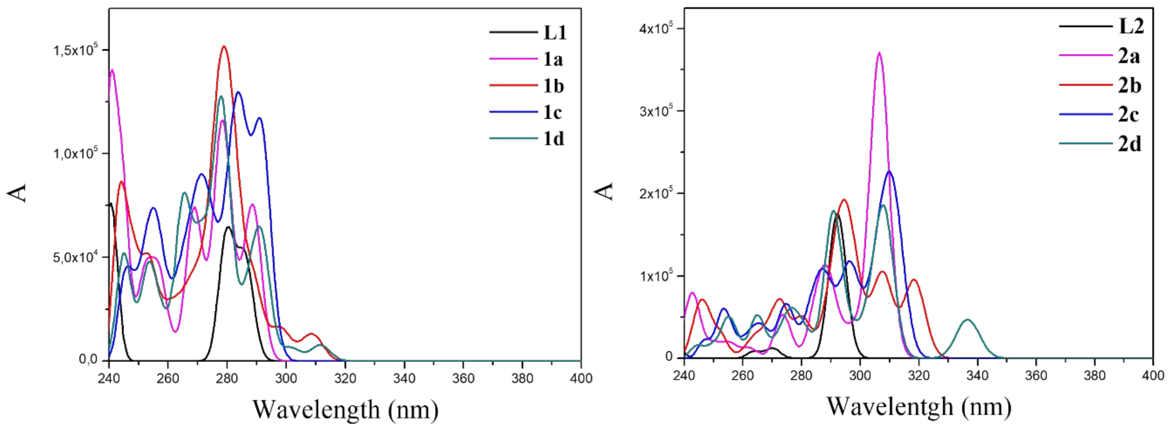

Figure S35. Calculated absorption spectra of dichloromethane solutions of **L1** (left) and **L2** derivatives (right).

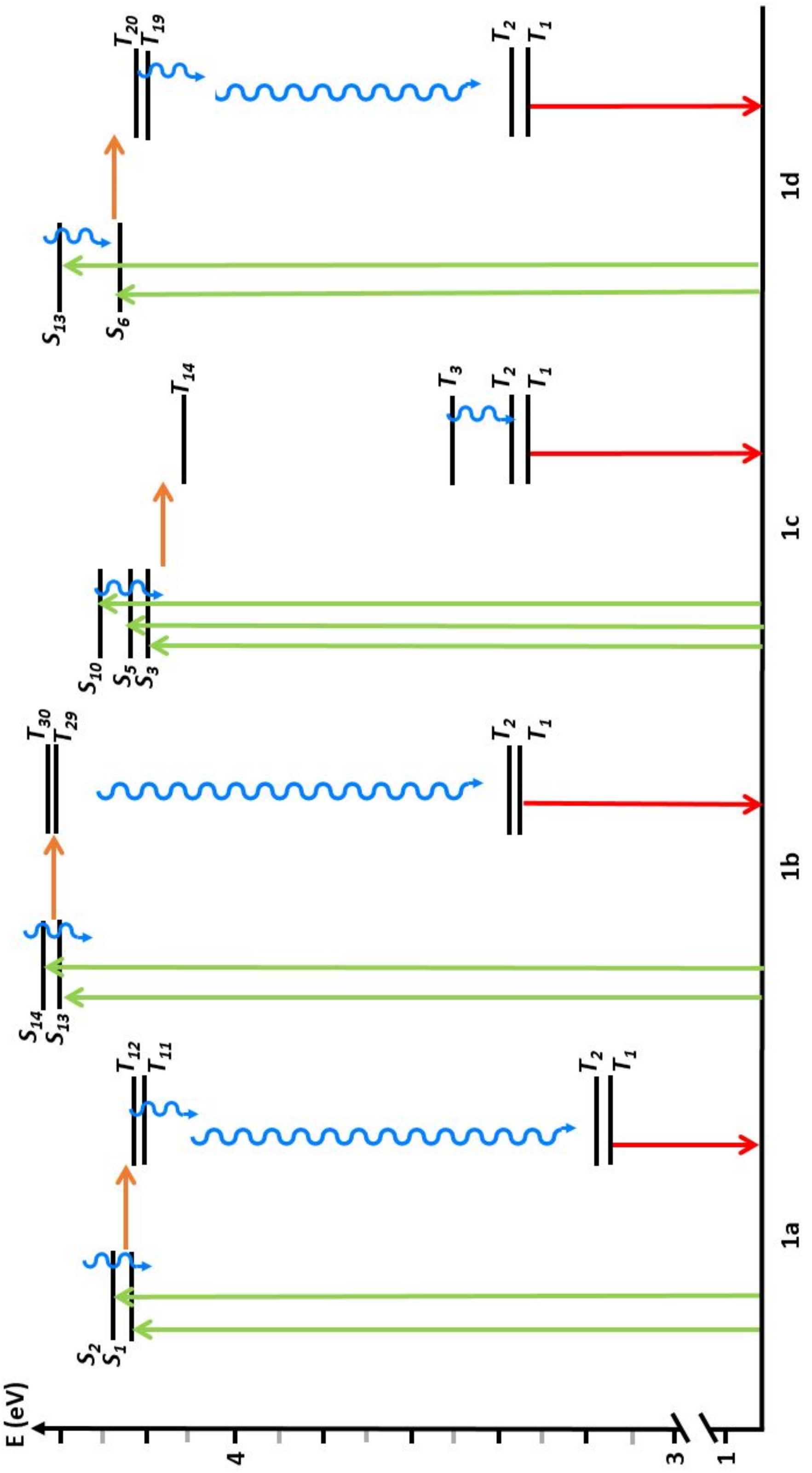

Scheme S1. TD-DFT state plot showing the energies of the S_1 and T_1 states in electronvolts for the **1x** family.

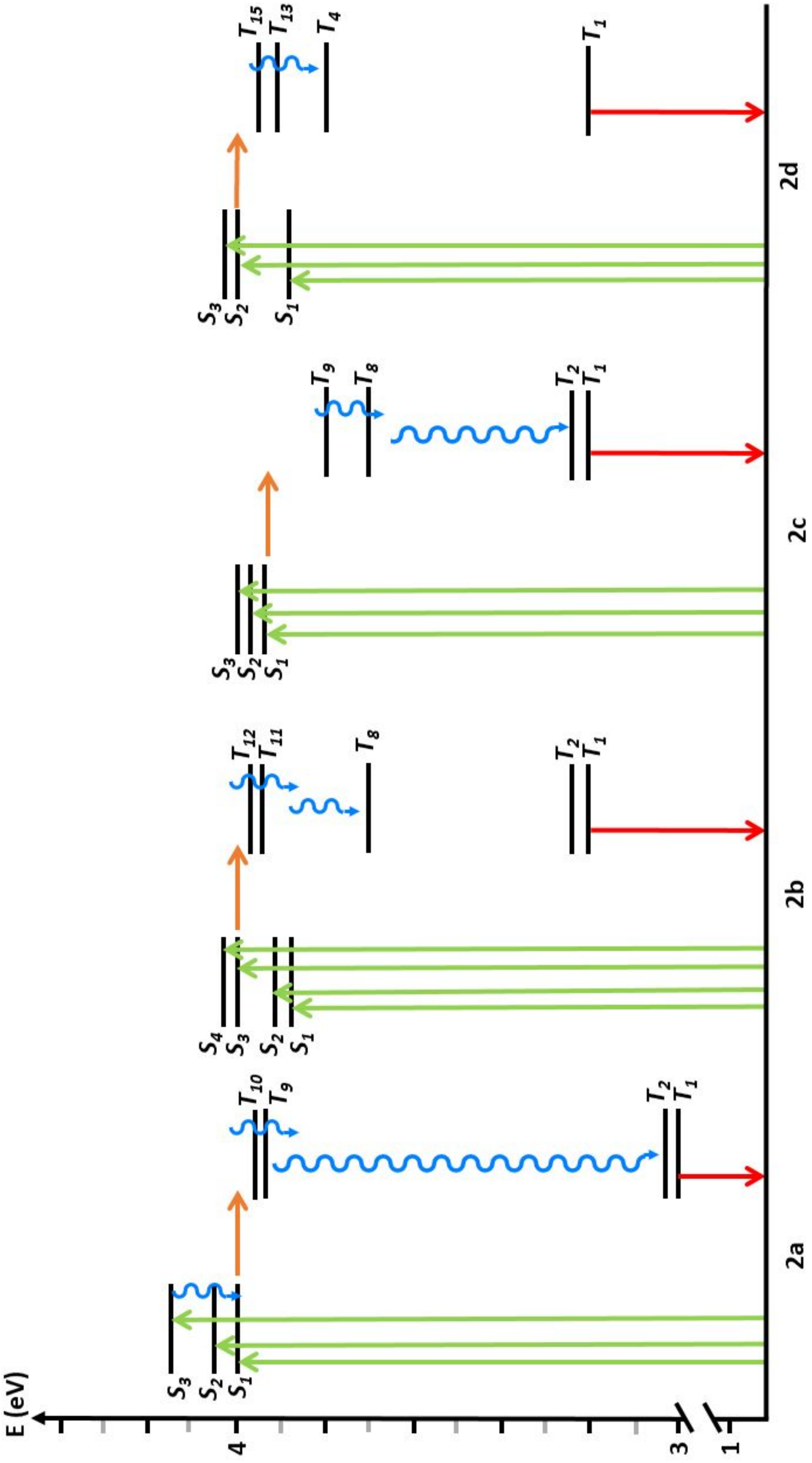

Scheme S2. TD-DFT state plot showing the energies of the S_1 and T_1 states in electronvolts for the **2x** family.

Table S1. The calculated S_n and T_n energies by TD-DFT for **1b** and **2b**, with their corresponding $S_1 \rightarrow T_n$ transition and its main contribution in %.

Compound	Energy (S_n) (eV)	Transitions	Energy (T_n) (eV)	Transitions
**1b**	S_{13}: 4.4024<br>f = 0.1668	H-5 → L (77%)<br>H → L+3 (5%)	T_{30}: 4.4226	H-1 → L+3 (34%)<br>H-2 → L+1 (8%)
	S_{14}: 4.4301<br>f = 0.1107	H-1 → L+3 (70%)<br>H-2 → L+1 (5%)	T_{29}: 4.4103	H-5 → L+1 (10%)<br>H-5 → L+2 (8%)<br>H-6 → L (8%)
			T_2: 3.1840	H → L+4 (17%)<br>H-1 → L+4 (16%)<br>H → L+2 (8%)
			T_1: 3.1814	H → L+3 (6%)<br>H-1 → L+3 (6%)
**2b**	S_1: 3.8797<br>f = 0.1638	H → L (95%)	T_{12}: 3.9189	H-1 → L+13 (16%)<br>H → L+14 (16%)<br>H-1 → L+14 (16%)
	S_2: 3.9053<br>f = 0.2028	H-1 → L (96%)	T_{11}: 3.9178	H → L+13 (17%)<br>H-1 → L+13 (15%)
	S_3: 4.0181<br>f = 0.1661	H → L+1 (89%)	T_8: 3.7263	H-18 → L+1 (4%)<br>H-10 → L+7 (3%)
	S_4: 4.0301<br>f = 0.1798	H-1 → L+1 (84%)	T_2: 3.0026	H-1 → L (8%)<br>H-1 → L+5 (25%)
			T_1: 3.0015	H → L (8%)<br>H-1 → L+1 (7%)

Table S2. The calculated S_n and T_n energies by TD-DFT for **1c** and **2c**, with their corresponding $S_1 \rightarrow T_n$ transition and its main contribution in %.

Compound	Energy (S_n) (eV)	Transitions	Energy (T_n) (eV)	Transitions
**1c**	S_3: 4.2388<br>f = 0.2253	H-1 → L+2 (22%)<br>H-3 → L+2 (14%)<br>H → L+1 (12%)<br>H → L (2%)	T_{14}: 4.1130	H-2 → L+14 (8%)<br>H → L+5 (6%)<br>H-1 → L+15 (5%)
	S_5: 4.2694<br>f = 0.1864	H-4 → L+2 (20%)<br>H-2 → L+2 (20%)<br>H → L+2 (8%)	T_3: 3.3687	H-5 → L (34%)<br>H-4 → L (4%)
	S_{10}: 4.3518<br>f = 0.2368	H-5 → L (22%)<br>H-4 → L (16%)<br>H → L (14%)	T_2: 3.1760	H-1 → L+2 (15%)<br>H-3 → L+2 (3%)
			T_1: 3.1725	H → L+2 (16%)<br>H-2 → L+2 (2%)
**2c**	S_1: 3.9625<br>f = 0.2741	H → L (46%)<br>H → L+1 (21%)<br>H-1 → L (16%)	T_9: 3.8571	H-3 → L (37%)<br>H-3 → L+1 (16%)<br>H-7 → L (8%)
	S_2: 3.9934<br>f = 0.1900	H → L (37%)<br>H-1 → L (29%)<br>H → L+2 (23%)	T_8: 3.7153	H-20 → L+2 (7%)<br>H-20 → L+3 (5%)<br>H-11 → L+2 (3%)
	S_3: 3.9948<br>f = 0.2424	H → L+1 (58%)<br>H-1 → L (21%)<br>H → L (4%)	T_2: 3.0010	H-1 → L (11%)<br>H-1 → L+6 (30%)
			T_1: 2.9997	H → L+2 (9%)<br>H → L+1 (2%)<br>H → L+4 (24%)

Table S3. The calculated S_n and T_n energies by TD-DFT for **1d** and **2d**, with their corresponding $S_1 \rightarrow T_n$ transition and its main contribution in %.

Compound	Energy (S_n) (eV)	Transitions	Energy (T_n) (eV)	Transitions
**1d**	S_6: 4.2503<br>f = 0.1048	H → L+1 (50%)<br>H-2 → L+1 (19%)<br>H-3 → L+2 (7%)	T_{20}: 4.2185	H-7 → L (18%)<br>H-8 → L (14%)<br>H-4 → L (11%)
	S_{13}: 4.4315<br>f = 0.1070	H-2 → L+1 (23%)<br>H-3 → L+1 (19%)<br>H-2 → L+2 (8%)	T_{19}: 4.2049	H-1 → L+16 (11%)<br>H-3 → L+20 (5%)
			T_2: 3.1814	H-1 → L+1 (7%)<br>H-1 → L+4 (12%)
			T_1: 3.1813	H → L+1 (3%)<br>H-1 → L+5 (12%)
**2d**	S_1: 3.6754<br>f = 0.1040	H → L (98%)	T_{15}: 3.9505	H-1 → L+2 (8%)<br>H-5 → L+13 (6%)
	S_3: 4.0181<br>f = 0.3526	H-1 → L+1 (70%)<br>H → L+2 (10%)	T_{13}: 3.9431	H-2 → L (74%)
	S_4: 4.0241<br>f = 0.2381	H → L+1 (89%)<br>H-1 → L+1 (3%)	T_4: 3.6127	H-1 → L (19%)<br>H-6 → L+6 (18%)
			T_1: 2.9960	H → L+2 (10%)<br>H → L (8%)<br>H-1 → L+1 (6%)

Table S4. Crystal data and structure refinement for **1a-d**.

Compound	1a	1b	1c	1d
Formula	$C_{52}H_{42}Au_2O_2P_2$	$C_{60}H_{42}Au_2O_3P_2$	$C_{63}H_{46}Au_2O_3P_2$	$C_{60}H_{42}Au_2O_2P_2$
Crystal size, mm	0.07 x 0.18 x 0.24	0.13 x 0.19 x 0.25	0.12 x 0.28 x 0.34	0.06 x 0.06 x 0.17
Fw	1154.73	1266.81	1306.87	1250.81
Temp., K	120.0(1)	120.0(1)	170(1)	120.0(1)
Wavelenght, Å	0.71073	0.71073	0.71073	1.54184
Crystal system	Triclinic	Monoclinic	Triclinic	Monoclinic
Space group	$P\bar{1}$	$C2/c$	$P\bar{1}$	$P2_1/n$
a, Å	12.8847(6)	45.2511(5)	11.1236(5)	12.3800(3)
b, Å	13.0105(7)	9.84320(10)	13.5215(4)	15.8576(3)
c, Å	16.2503(4)	21.8338(3)	19.0217(9)	24.2889(5)
α, °	68.727(4)	90	96.973(2)	90
β, °	74.096(3)	97.6550(10)	103.574(2)	100.293(2)
γ, °	89.373(4)	90	111.619(2)	90
Volume, $Å^3$	2429.4(2)	9638.4(2)	2516.13(18)	4691.59(16)
Z	2	8	2	4
$D_{calc.}$, mg m^{-3}	1.579	1.746	1.725	1.771
Abs. coef., mm^{-1}	6.134	6.195	5.935	12.583
F(000)	1116	4912	1272	2424
θ range for data coll, °	2.232 to 29.053	2.196 to 27.101	1.655 to 27.096	4.329 to 76.1060
Refins coll./independent	18962/11022	33066/10514	18780/10975	17535/9152
Data/restraint/parameters	11022/91/557	10514/0/604	10975/0/633	9152/0/595
GOF on F^2	1.021	1.047	1.070	1.026
Final R index ($I > 2\sigma(I)$)	R_1 = 0.0350, wR_2 = 0.0735	R_1 = 0.0242, wR_2 = 0.0438	R_1 = 0.0346, wR_2 = 0.0884	R_1 = 0.0412, wR_2 = 0.1003
R index (all data)	R_1 = 0.0470, wR_2 = 0.0798	R_1 = 0.0310, wR_2 = 0.0461	R_1 = 0.0544, wR_2 = 0.1005	R_1 = 0.0532, wR_2 = 0.1101
Peak and hole, e $Å^{-3}$	2.26 and -1.51	0.63 and -0.55	0.71 and -1.17	5.46 and -1.42
CCDC	2193612	2193613	2193614	2193615

Table S5. Crystal data and structure refinement for **2a-d**.

Compound	2a	2b	2c	2d
Formula	$C_{58}H_{54}Au_2P_2$	$C_{66}H_{54}Au_2OP_2$	$C_{69}H_{58}Au_2OP_2 \cdot CH_2Cl_2$	$C_{66}H_{54}Au_2P_2$
Crystal size, mm	0.02 x 0.05 x 0.24	0.01 x 0.05 x 0.14	0.02 x 0.07 x 0.12	0.19 x 0.25 x 0.37
Fw	1206.88	1318.96	1443.95	1302.96
Temp., K	120.0(1)	120.0(1)	120.0(1)	120.0(1)
Wavelenght, Å	0.71073	0.71073	1.54184	0.71073
Crystal system	Triclinic	Monoclinic	Monoclinic	Orthorhombic
Space group	$P\bar{1}$	$P2_1/n$	$P2_1/n$	*Pbcn*
a, Å	15.7858(3)	16.6966(3)	10.5875(2)	16.7034(3)
b, Å	16.2186(5)	12.4413(4)	29.2014(4)	13.2625(2)
c, Å	20.4661(4)	26.0271(6)	18.9250(3)	24.3421(3)
α, °	71.180(2)	90	90	90
β, °	80.030(2)	95.995(2)	101.948(2)	90
γ, °	86.445(2)	90	90	90
Volume, $Å^3$	4884.7(2)	5377.0(2)	5724.28(17)	5392.48(14)
Z	4	4	4	4
$D_{calc.}$, mg m^{-3}	1.641	1.629	1.675	1.605
Abs. coef., mm^{-1}	6.102	5.553	11.228	5.535
F(000)	2360	2584	2840	2552
θ range for data coll, °	2.662 to 28.332	2.487 to 27.492	2.799 to 76.299	2.573 to 28.920
Refins coll./independent	52304/21176	23027/10981	21387/11154	13634/5517
Data/restraint/parameters	21176/0/1125	10981/0/644	11154/0/700	55117/72/346
GOF on F^2	1.038	0.986	1.051	1.207
Final *R* index ($I > 2\sigma(I)$)	R_1 = 0.0465, wR_2 = 0.0926	R_1 = 0.0481, wR_2 = 0.0733	R_1 = 0.0296, wR_2 = 0.0614	R_1 = 0.0420, wR_2 = 0.0833
R index (all data)	R_1 = 0.0797, wR_2 = 0.1086	R_1 = 0.0879, wR_2 = 0.0857	R_1 = 0.0424, wR_2 = 0.0671	R_1 = 0.492, wR_2 = 0.0860
Peak and hole, e $Å^{-3}$	6.69 and -2.48	1.11 and -1.08	0.96 and -0.99	2.07 and -1.71
CCDC	2193616	2193617	2193618	2193619
